# *In Vitro* Expansion of Keratinocytes on Human Dermal Fibroblast-Derived Matrix Retains Their Stem-Like Characteristics

**DOI:** 10.1038/s41598-019-54793-9

**Published:** 2019-12-06

**Authors:** Chee-Wai Wong, Catherine F. LeGrand, Beverley F. Kinnear, Radoslaw M. Sobota, Rajkumar Ramalingam, Danielle E. Dye, Michael Raghunath, E. Birgitte Lane, Deirdre R. Coombe

**Affiliations:** 1 School of Pharmacy and Biomedical Sciences, Faculty of Health Sciences, Curtin University, Bentley, WA 6102 Australia; 2 Curtin Health Innovation Research Institute, Faculty of Health Science, Curtin University, Bentley, WA 6102 Australia; 3 Institute of Molecular and Cell Biology, Agency for Science, Technology and Research (A*STAR), 61 Biopolis Drive, No. 07-48A Proteos, Singapore, 138673 Singapore; 4 Skin Research Institute of Singapore and Institute of Medical Biology, Agency for Science, Technology and Research (A*STAR), 8A Biomedical Grove, 06-06 Immunos, Singapore, 138648 Singapore; 5 Centre for Cell Biology and Tissue Engineering, Competence Centre for Tissue Engineering and Substance Testing (TEDD), Institute for Chemistry and Biotechnology, ZHAW School of Life Science and Facility Management, Zurich University of Applied Science, Winterthur, Switzerland; 6 Centre for Cell Therapy and Regenerative Medicine, School of Biomedical Sciences, The University of Western Australia, Crawley, WA Australia

**Keywords:** Regenerative medicine, Skin stem cells

## Abstract

The long-term expansion of keratinocytes under conditions that avoid xenogeneic components (i.e. animal serum- and feeder cell-free) generally causes diminished proliferation and increased terminal differentiation. Here we present a culture system free of xenogeneic components that retains the self-renewal capacity of primary human keratinocytes. *In vivo* the extracellular matrix (ECM) of the tissue microenvironment has a major influence on a cell’s fate. We used ECM from human dermal fibroblasts, cultured under macromolecular crowding conditions to facilitate matrix deposition and organisation, in a xenogeneic-free keratinocyte expansion protocol. Phospholipase A_2_ decellularisation produced ECM whose components resembled the core matrix composition of natural dermis by proteome analyses. Keratinocytes proliferated rapidly on these matrices, retained their small size, expressed p63, lacked keratin 10 and rarely expressed keratin 16. The colony forming efficiency of these keratinocytes was enhanced over that of keratinocytes grown on collagen I, indicating that dermal fibroblast-derived matrices maintain the *in vitro* expansion of keratinocytes in a stem-like state. Keratinocyte sheets formed on such matrices were multi-layered with superior strength and stability compared to the single-layered sheets formed on collagen I. Thus, keratinocytes expanded using our xenogeneic-free protocol retained a stem-like state, but when triggered by confluence and calcium concentration, they stratified to produce epidermal sheets with a potential clinical use.

## Introduction

The skin is an indispensable barrier that safeguards the body from the external environment. It possesses the ability to self-renew, which enables the replacement of dead cells and the repair of wounds, thereby sustaining a barrier function^1^. In normal circumstances, most cutaneous wounds heal without medical intervention. However, if the wound is extensive and extends into the dermis, medical attention may be required^2^. Traditionally, the therapeutic strategy for treating large, deep wounds has been to use split-thickness skin autografts. However, this treatment is not viable in the case of extensive burn injury, as patients may lack sufficient healthy donor sites^3^.

The grafting of cultured keratinocytes is an alternative treatment to assist in the repair of damaged skin. This method uses a technique originally developed by Rheinwald and Green^4^ to expand keratinocytes *in vitro* from a patient’s skin biopsy. The expansion of keratinocytes is achieved using an irradiated mouse fibroblast feeder layer and medium containing foetal bovine serum (FBS). While this method is effective for rapidly expanding keratinocytes, the reliance on xenogeneic components carries a potential risk of exposing the patients to animal pathogens and immunogenic molecules^5^. To address these concerns, *in vitro* culture systems that omit both the feeder layer and serum have been developed, including a popular system that uses a defined serum-free medium containing the necessary growth factors and a collagen matrix to support keratinocyte attachment and growth^6,7^.

However, keratinocytes grown in this defined serum-free system have a more limited lifespan, with diminished self-renewal capacity and an increased commitment towards differentiation or senescence^7,8^, compared to keratinocytes cultured using the Rheinwald and Green^4^ system. This suggests that defined serum-free medium and a collagen matrix do not fully meet keratinocyte requirements. It is likely that crucial elements required to sustain undifferentiated keratinocytes long-term reside in the fibroblast feeders used in the Rheinwald and Green system. Fibroblasts secrete cytokines, growth factors and extracellular matrix (ECM). The focus for defined culture systems has been on the cytokines and growth factors^9,10^, but the ECM is also a crucial requirement that has received much less attention.

The ECM is complex meshwork of macromolecules, comprising fibrous structural proteins (e.g. collagen, fibronectin, laminin and elastin), specialised proteins (e.g. growth factors) and proteoglycans (e.g. perlecan). It was previously thought to be an inert structure that provided a platform for cell adhesion, but it is now known that the ECM also provides both biochemical and biomechanical cues that regulate cell behaviours like adhesion, migration, proliferation and differentiation^11,12^. Currently, there is considerable interest in using cell-derived matrices to reproduce the cells’ microenvironment as it is found in tissues. Numerous studies have shown that acellular ECM assists in maintaining the stem cell phenotype and in promoting self-renewal during *in vitro* expansion^13–16^. However, the effect of a fibroblast derived-matrix on keratinocyte proliferation in the absence of serum has not been examined.

While it is possible to generate an acellular ECM *in vitro*, most *in vitro* culture methods produce an unstructured ECM that lacks critical components such as collagens and proteoglycans^17,18^. It is possible that differences between the *in vitro* and *in vivo* microenvironments contribute to the less structured ECM that is produced in tissue culture. Cells in culture are in a dilute solution of macromolecules (i.e. proteins and lipids) of around 1–10 mg/ml, which is several-fold lower than the normal physiological environment that can range from 20.6 mg/ml to 80 mg/ml^19^. Thus, in culture, molecular interactions taking place outside of cells may not be occurring at rates required for the assembly of an optimal ECM. To mitigate this problem, the addition of large, inert macromolecules to the culture medium has been used to better mimic the density of macromolecules within tissues, a process called “macromolecular crowding” (MMC). Ficoll is a large, neutral, hydrophilic polysaccharide that dissolves in aqueous solutions, and when used in this context, is described as a “macromolecular crowder”. The addition of Ficoll to cell cultures has been found to accelerate biochemical reactions and supramolecular assembly, and macromolecular crowding has been found to positively affect the deposition and architecture of the ECM^17,18,20^. We have previously applied MMC to enhance the *in vitro* deposition of ECM by dermal fibroblasts, to accelerate the development of skin organotypic cultures^21^.

**Figure 1:**
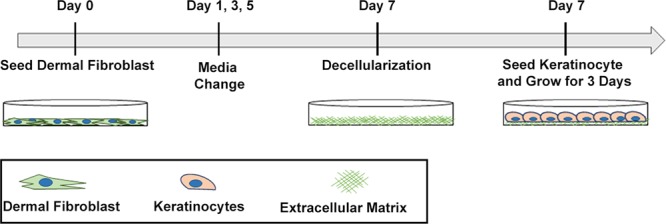
Schematic of the preparation of a xenogeneic-free acellular dermal fibroblast-derived matrix as a substrate for keratinocyte expansion.

Here, we describe the development and functional characterization of a xenogeneic-free matrix derived from primary human dermal fibroblasts under MMC conditions (Fig. 1). Proteomic analyses by mass spectrometry confirmed that this matrix resembled, in its core protein composition, the ECM of human dermal tissue. When used as a substrate for keratinocyte growth in the absence of feeder cells and under defined serum-free conditions, this fibroblast-derived matrix was found to facilitate keratinocyte proliferation. More keratinocytes maintained the stem-like characteristics of small cell size, expression of p63 and a lack of keratin 16 expression, as well as the retention of a colony forming capability when they were grown on fibroblast-derived matrix, compared to tissue culture plastic or collagen I. These data indicated that these acellular fibroblast-derived matrices provide an appropriate microenvironment to enable the expansion of undifferentiated keratinocytes *in vitro*. Importantly, keratinocytes grown to confluence on these matrices, when triggered by the appropriate calcium concentration, retained the ability to form multilayered, stratified epidermal sheets resistant to considerable mechanical stress. Such cell sheets have the characteristics required for clinical use, although this aspect was not specifically examined in this study.

## Results

### Development of an Acellular Dermal Fibroblast-Derived Matrix

To produce a matrix which best mimics the microenvironment that keratinocytes would encounter *in vivo*, primary human dermal fibroblasts (HDFs) from adult donors were chosen as the cell source. Phase contrast microscopy revealed that the HDFs selected had a uniform spindle-like morphology that is typical of fibroblasts. Immunofluorescence analyses indicated they expressed the fibroblast markers TE-7, Thy-1 and vimentin (Fig. S1).

**Figure 2:**
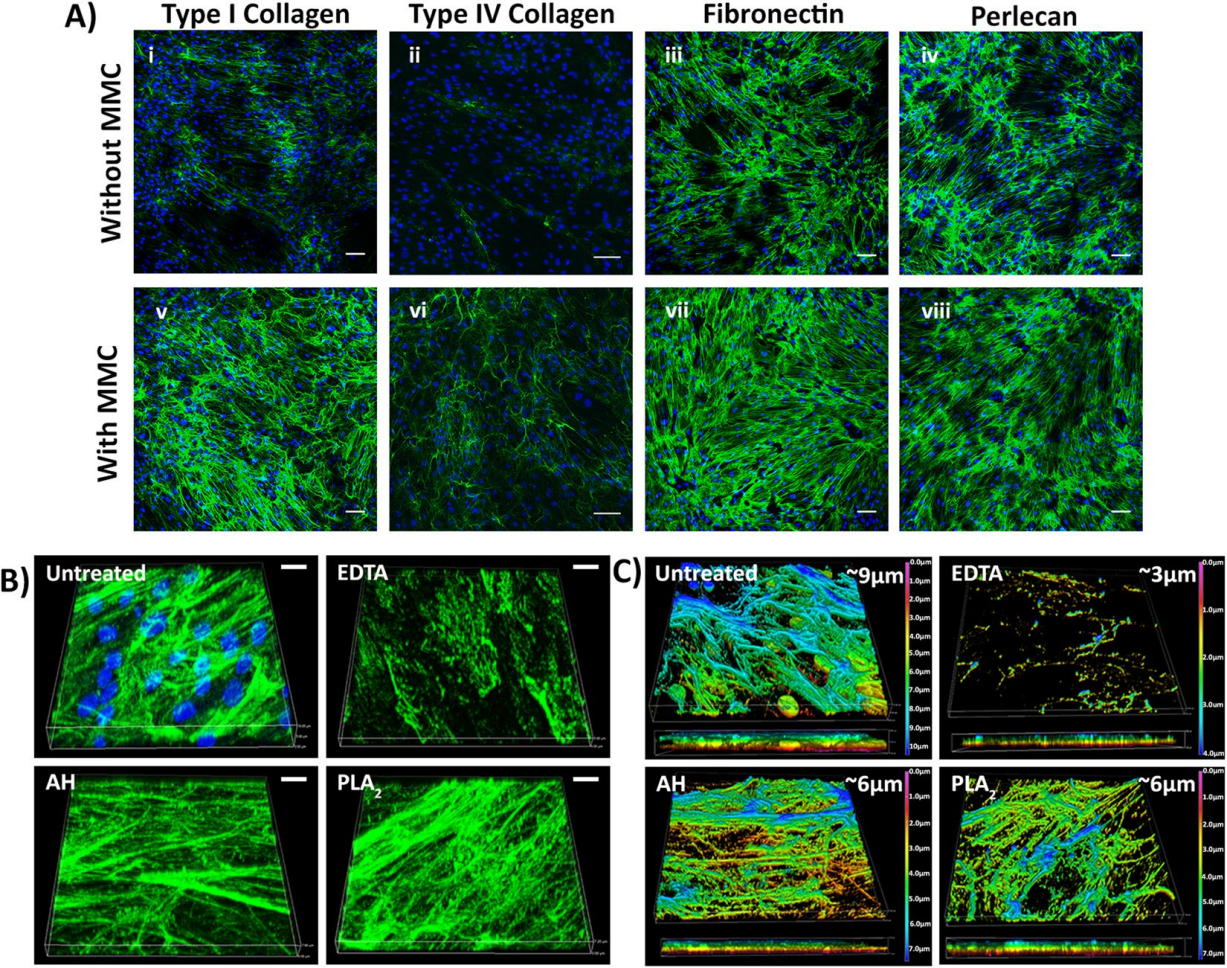
ECM proteins deposited by dermal fibroblasts (**A**) The effect of MMC on HDF ECM deposition. The cells and matrix were immunostained for collagen I (i, v), collagen IV (ii, vi), fibronectin (iii, vii) and perlecan (iv, viii). Scale bars are 100 µm. (**B**) The 3D architecture of the ECM deposited by HDF before and after decellularisation with EDTA, ammonium hydroxide (AH) or phospholipase A_2_ (PLA_2_) as revealed by collagen I immunostaining and DAPI staining. Scale bars are 10 µm. (**C**) The thickness of the ECM after decellularisation. The colour coding represents the Z-depth location within the 3D z-stacked image. Shown are data for ATCC PCS-201-012 ECM, but EBL028 derived ECM was of a similar thickness of 4–5 µm.

It has been reported that the addition of a mixture of Ficoll 70 and Ficoll 400 to the culture medium, which has the effect of mimicking how glycoproteins in plasma occupy space^17,21^, benefits ECM deposition by cells *in vitro*. Accordingly, the Ficoll MMC cocktail was included in the fibroblast culture medium. The addition of MMC marginally altered the appearance of the HDFs, and reduced their proliferation over a 7 day period (Fig. S2A,C), but their lack of immunoreactivity with an α-SMA antibody indicated they had not differentiated into myofibroblasts. In contrast, in the absence of Ficoll, α-SMA staining was detected and cell morphology of the α-SMA-positive cells resembled that of myofibroblasts (Fig. S2B). Immunostaining of the ECM deposited by HDFs cultured under MMC revealed increased staining intensities for collagens I and IV, fibronectin and perlecan compared to cultures without MMC (Fig. 2A). Only under MMC, deposited ECM appeared to fully surround and embed individual cells, as revealed in cross-sectional analyses of confocal microscopy images (Fig. S3A). Removal of the HDFs using a phospholipase A_2_ (PLA_2_) decellularisation protocol revealed a uniform deposition of collagen I and fibronectin across the surface when HDFs were cultured with MMC (Fig. S3B); and this was equally true for fibroblasts from two different donors (data for ATCC PCS-201-012 are shown, data for EBL028 not shown).

As MMC led to a well-structured and uniform deposition of ECM, without myofibroblast differentiation, this deposition system was used to generate the fibroblast-derived matrices, or Fib-Mats. To determine a decellularisation method which removed the cells yet preserved the ECM proteins and structure best, three protocols were compared: EDTA, ammonium hydroxide (AH) and PLA_2_. Phase contrast microscopy revealed that all three protocols removed the fibroblasts, however, fibril-like structures were retained only with AH and PLA_2_ treatments (Fig. S4). DAPI staining indicated both the EDTA and the PLA_2_ methods removed DNA remnants more effectively than the AH method, which left distinct nuclear fragments in the ECM (Fig. S5Ai). Quantification of DNA removal indicated that the EDTA and PLA_2_ methods were effective in removing 99% of the DNA, versus 97% of the DNA removed with the AH method (Fig. S5Aii). To determine whether the cytoskeletal components of the HDFs were removed after the decellularisation treatments, phalloidin-TRITC staining was used to detect actin filaments. As shown in Fig. S5B, a few actin filament fragments were detected following AH treatment, but no residual phalloidin staining was visible after EDTA or PLA_2_ treatments.

To investigate the structure of the ECM following decellularisation, 3-dimensional (3D) Z-stacked confocal images were obtained following collagen I immunostaining. Fibrillar structures of collagen I resembling the non-decellularised control were clearly visible following AH and PLA2 treatments, but the EDTA treatment disrupted the structure of the collagen I filaments (Fig. 2B). The thickness of the ECM as determined by the depth of collagen I staining, calculated from Z-stacked images obtained by confocal microscopy, was found to be 9 µm. After decellularisation using the AH or PLA_2_ protocols, ECM thickness decreased to around 6 µm, and following EDTA treatment it was 3 µm (Fig. 2C). Immunofluorescence intensities of the decellularised matrices revealed a significant reduction in collagen I and fibronectin staining after EDTA treatment. In contrast, both AH and PLA_2_ treatments were shown to preserve collagen I and fibronectin immunostaining (Fig. S6).

### Cultured dermal fibroblast matrices mimic native skin dermal ECM

**Figure 3:**
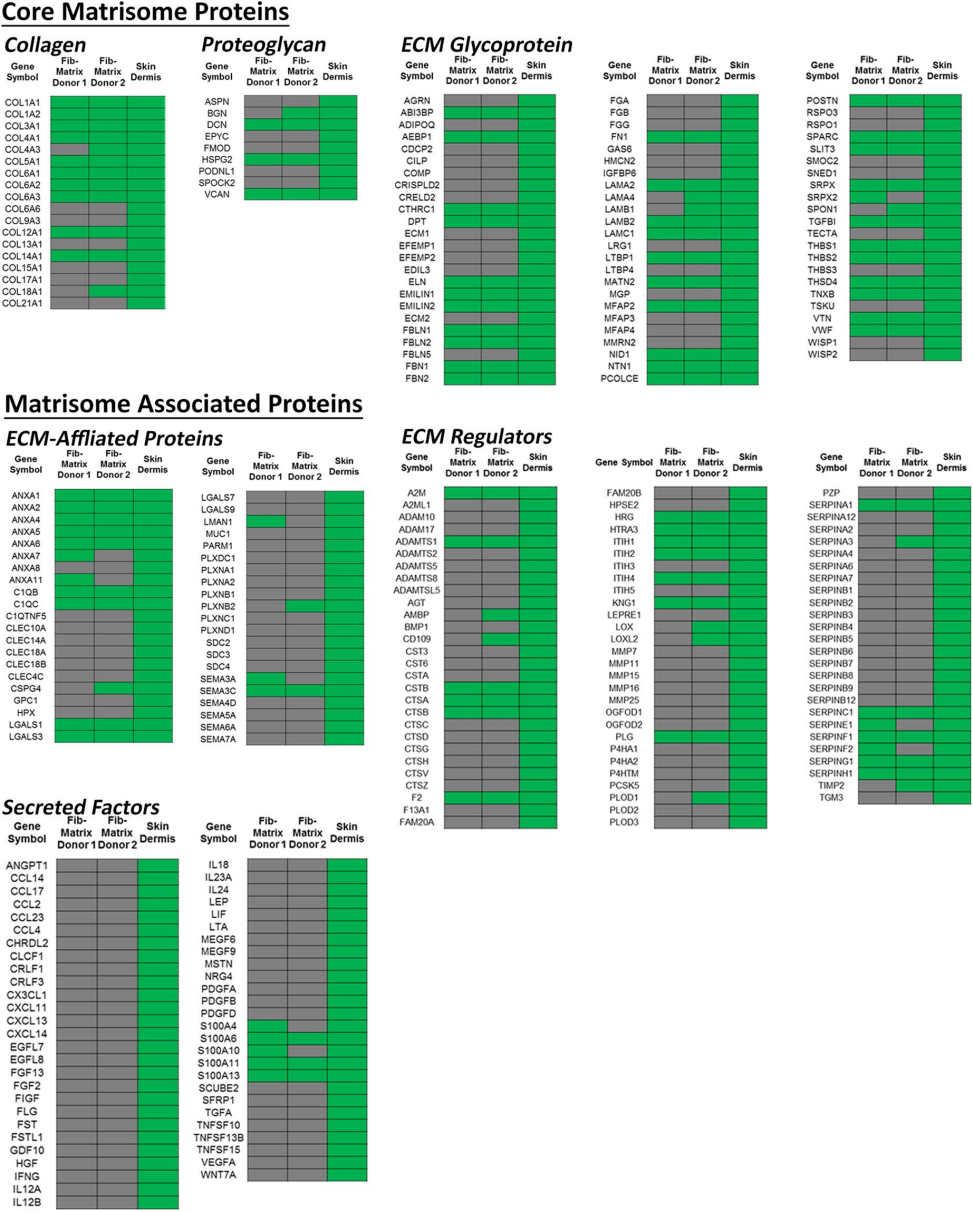
Protein composition of the acellular ECM. Protein compositions of the acellular ECM from dermal fibroblasts from two donors, obtained after culturing the fibroblasts under MMC, were compared to that of the dermis. The ECM proteins are subdivided into two categories; “core matrisome” (ECM glycoproteins, collagens and proteoglycans); and “matrisome-associated proteins” (ECM-affiliated proteins, ECM regulators and secreted factors). Green and grey indicate the presence or absence of ECM proteins respectively. Donor 1 cells = ATCC PCS-201-012, Donor 2 cells = EBL028.

Collectively the data demonstrated that the PLA_2_ decellularisation protocol produced an intact ECM that was devoid of most cell components, hence this method was used to generate the acellular Fib-Mats. Because our goal was the production of an acellular matrix that mimicked the *in vivo* dermal ECM, the protein compositions of the ECM from two different dermal fibroblast donors were determined using mass spectrometry (MS)-based proteomics. We then compared the data with the ECM signature of human dermis as given in the Human Protein Atlas database^22^. When we realized that the Human Protein Atlas does not contain a number of core dermal ECM proteins like collagen III alpha 1 (COL3A1) and laminin alpha-4 (LAMA4), we combined the Atlas with the proteomic dataset of skin prepared from studies by Bliss *et al*.^23^. To curate the combined dermal ECM protein list we categorised it using the human matrisome database (MatrisomeDB, http://matrisomeproject.mit.edu/). This database classifies ECM proteins as “core matrisome” (ECM glycoproteins, collagens and proteoglycans) or “matrisome-associated proteins” (ECM-affiliated proteins, ECM regulators and secreted factors)^24^. This analysis revealed that most of the core matrisome proteins expressed in the skin dermis were also found in the Fib-Mats generated under MMC (Fig. 3). However, most of the matrisome-associated proteins were not detected in the Fib-Mats (Fig. 3). The compositions of the Fib-Mats prepared from the two different adult fibroblast populations were very similar.

### Dermal fibroblast-derived matrix supports keratinocyte proliferation

**Figure 4:**
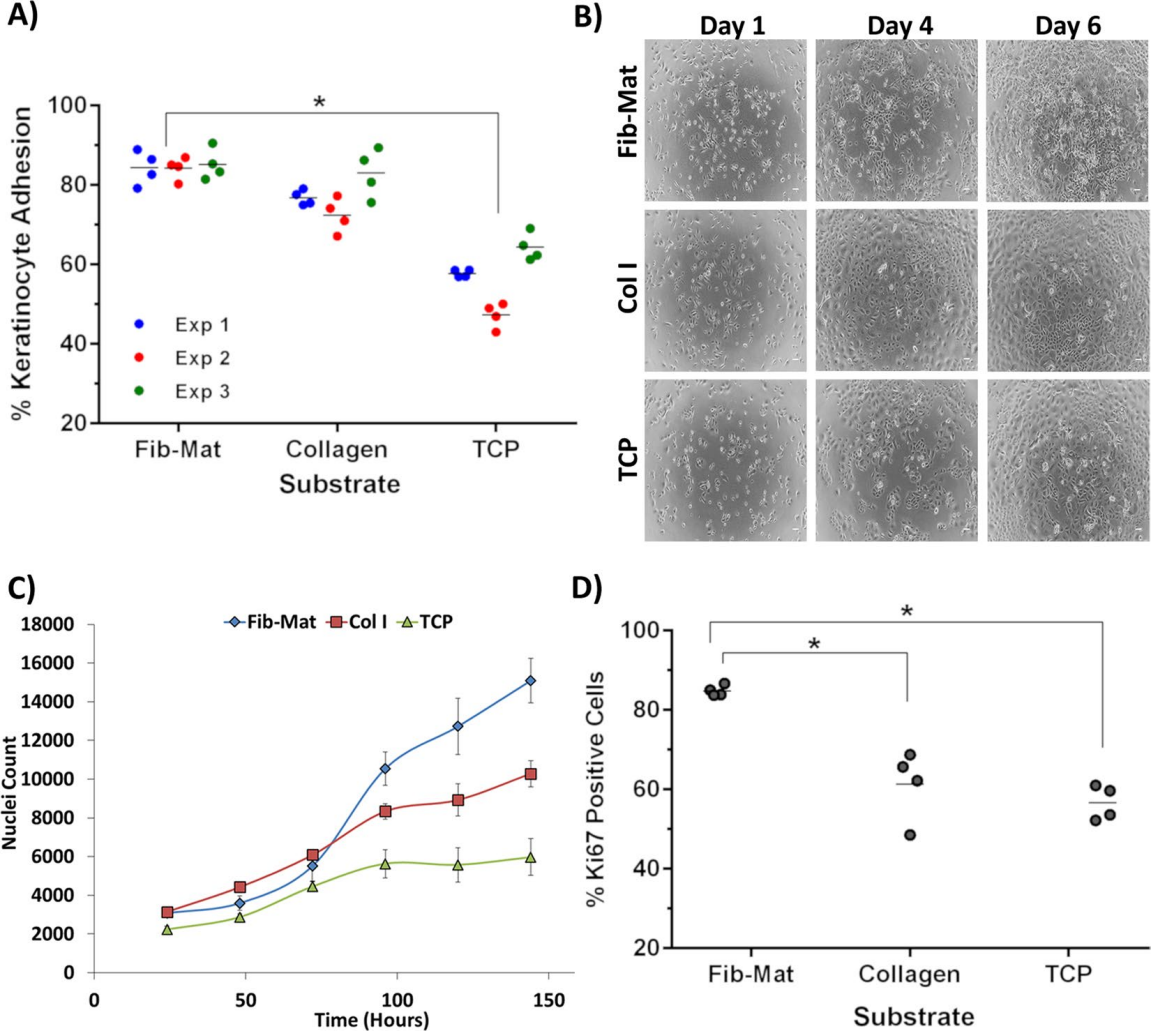
The different substrates support keratinocyte adhesion and proliferation to varying degrees. (**A**) The ability of Fib-Mat, Collagen I and TCP to support keratinocyte adhesion. Shown are data from three separate experiments, where each data point is the percent of bound keratinocytes per well; means are shown as a line. *p < 0.01 (**B**) Morphology of keratinocytes growing on dermal fibroblast-derived matrix (Fib-Mat), collagen I (Col I) and tissue culture plastic (TCP) as captured by phase contrast microscopy. Keratinocyte on days 1, 4 and 6 post seeding are shown. Scale bars are 100 μm. (**C**) The ability of Fib-Mat, Col I and TCP to support keratinocyte proliferation. Nuclei were stained with DAPI and counted. The data are from 4 replicate wells of three separate experiments. Means are shown. (**D**) Ki67 expression by keratinocytes cultured on Fib-Mat, Col I and TCP on day 3. The data are the percent of Ki67 positive keratinocytes for each of 4 replicate wells; mean values are shown as a line. These data are representative of three separate experiments. *p < 0.01.

**Figure 5:**
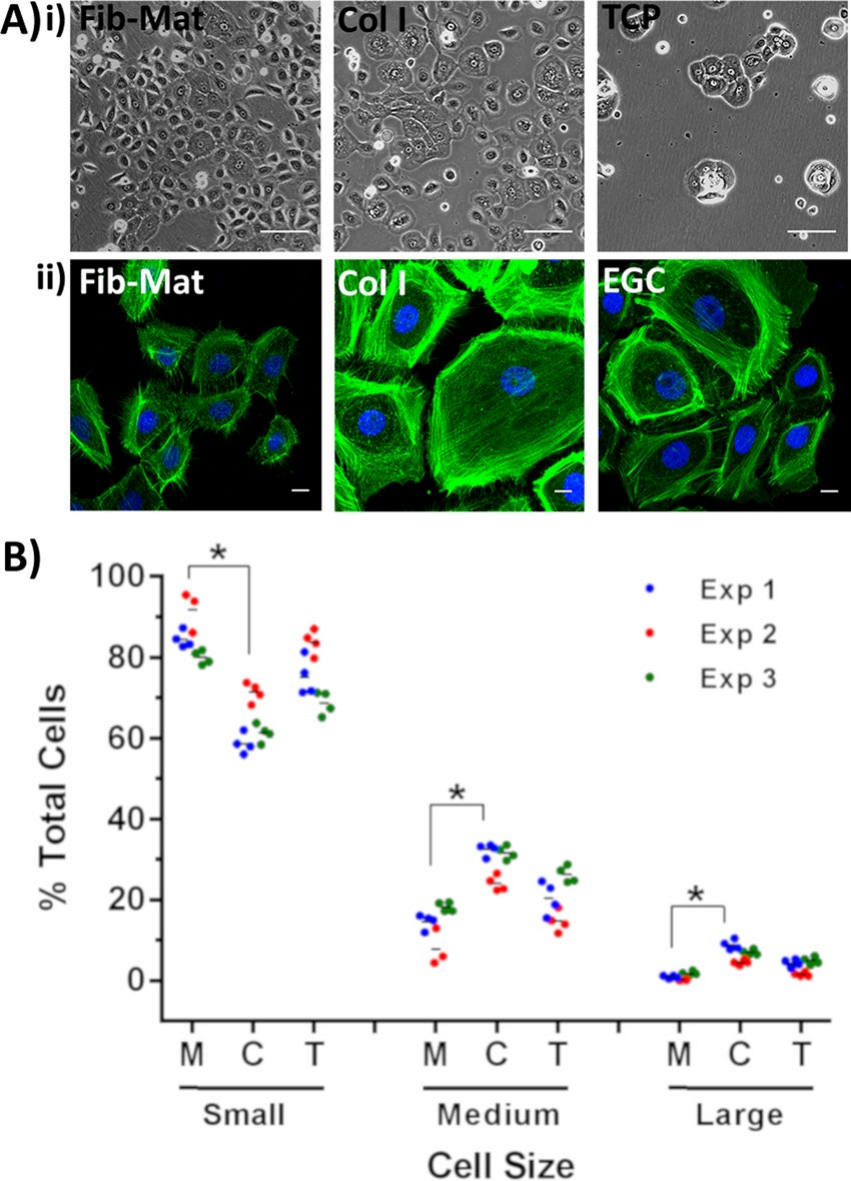
Size of keratinocytes grown on different substrates. (**A**) (i) Phase contrast images show differences in the size of keratinocytes grown on the various substrates. Images were captured on day 3 of culture. Scale bars are 100 µm. (ii) Representative images of keratinocytes stained with phalloidin-Alexa Fluor 488 demonstrating that phalloidin staining accurately revealed keratinocyte size. Scale bars are 50 μm. Nuclei were stained using DAPI (Blue). (**B**) Frequency of keratinocytes of differing size. Cells were assigned into categories based on cell area (small < medium < large = <2000 μm^2^ < 4000 μm^2^ < 6000 μm^2^), where M = FibMat, C = Col I and T = TCP. The data are the percent of total cells.  Data from three separate experiments using Fib-Mats from ATCC PCS-201-012 are shown. *p ≤ 0.01, but replicate experiments using Fib-Mats from EBL028 gave very similar data.

The ability of Fib-Mats to support the adhesion and proliferation of keratinocytes was investigated. Collagen I was used as a positive control, as it is the substrate recommended for use with the defined keratinocyte serum-free medium (DKSFM; Gibco) which we used to grow the keratinocytes^6,7^. A substrate lacking matrix proteins, tissue culture plastic (TCP), was the negative control and in DKSFM cultures serum fibronectin, vitronectin or other serum matrix proteins were not deposited on the TCP. The extent of keratinocyte adhesion to the various substrates differed. Quantification of adhesion revealed that significantly more keratinocytes attached to Fib-Mat (84%) than to TCP (56%; Fig. 4A). This is consistent with phase contrast microscopy on day 1, which revealed that keratinocytes adhered well to both Fib-Mat and collagen I but poorly to TCP (Fig. 4B). While keratinocytes proliferated on all three substrates, their behaviour differed. On Fib-Mat the keratinocytes grew as colonies, and cells within the colonies were small with a cobblestone morphology, which persisted until day 6 whereupon the cells began to reach confluence. Although similar behaviour was observed on TCP, the keratinocytes comprised a heterogeneous population of differing sizes. Whilst keratinocytes on collagen I grew as single cells, they also developed mixed morphologies and some of the cells were quite large and flat (Figs. 4B and 5A).

The rate of keratinocyte proliferation on the various substrates also differed. On Fib-Mat, keratinocytes initially proliferated more slowly than keratinocytes on collagen I, but reached a similar cell density by day 3. Thereafter, an exponential rate of proliferation was observed on Fib-Mat, which was higher than that seen on collagen I. On TCP, keratinocyte proliferation was slower and the rate plateaued by day 4 (Fig. 4C). Keratinocyte expression of Ki67 was determined on day 3, as the growth curve (Fig. 4C) indicated a change in proliferation rates on each of the substrates at this point. More keratinocytes were stained with the Ki67 mAb on Fib-Mat (84.85%) than on collagen I (66.31%) or TCP (56.66%). This difference was significant (p < 0.01) (Fig. 4D). Determination of the numbers of Ki67-expressing cells on day 4 and 5 revealed that this significant difference persisted (data not shown). From these data, Fib-Mat emerged as the most suitable substrate to promote keratinocyte proliferation.

### A higher proportion of keratinocytes grown on dermal fibroblast-derived matrix remain undifferentiated

**Figure 6:**
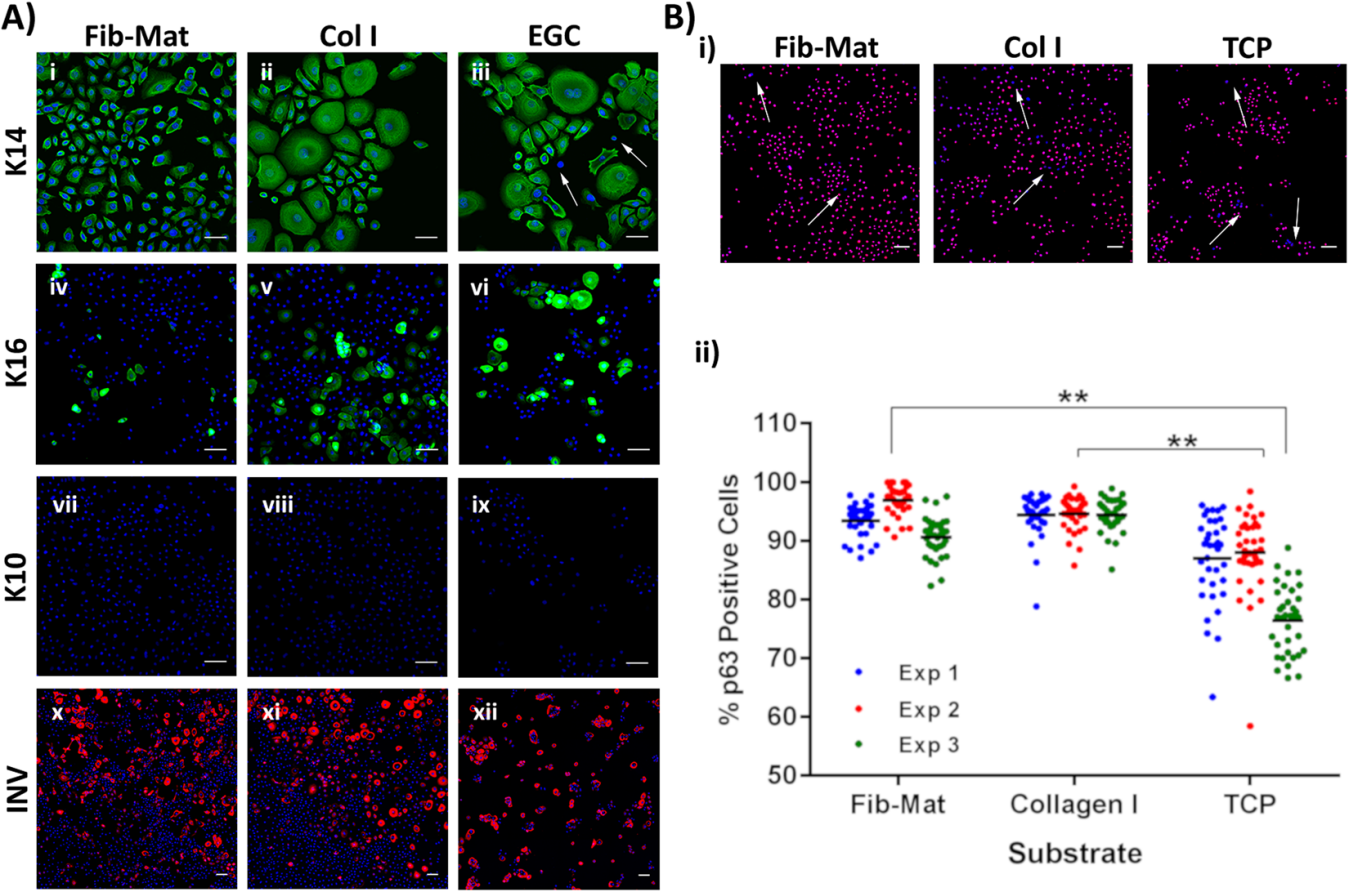
Expression of differentiation markers by keratinocytes grown on different substrates. (**A**) The expression of K14 (i-iii), K16 (iv-vi), K10 (vii-ix) and involucrin (x-xii) by keratinocytes grown for three days on Fib-Mat, Col I and ECG. Nuclei were stained using DAPI (Blue). Scale bars are 50 μm for K14 and 100 μm for K10, K16 and involucrin. Arrows indicate keratinocytes with no K14 expression. (**B**) Expression of p63 by keratinocytes grown on Fib-Mat, Col I and TCP. i) Representative image of keratinocytes immunostained for p63. Arrows indicate the area of p63 negative keratinocytes. The nuclei were stained using DAPI (Blue) Scale bars are 100 μm. ii) Quantification of keratinocytes positive for p63. The data shown are from three separate experiments. Mean values are shown as a line. **p < 0.001

Keratinocytes grown on the different substrates were assessed for indicators of terminal differentiation using antibodies to keratin 10 (K10), involucrin, keratin 16 (K16), and as a control keratin 14 (K14). To acquire better image resolution at high magnification, etched glass coverslips (EGC) were used to support the cultures. To ascertain that this growth surface did not affect keratinocyte behaviour, keratinocytes were grown on either TCP or EGC, which were coated with Fib-Mat or collagen I, or were uncoated. Cells were grown for 3 days before being fixed and immunostained for K14. Keratinocytes were similarly positive for K14 on all surfaces (Fig. S7). On collagen I coated EGC, keratinocytes grew as colonies, whereas they grew as single cells on collagen I coated TCP. As this was the only change, immunostaining of the differentiation markers was performed on keratinocytes grown on EGC with or without the various coatings. Although K14 expression was observed in keratinocytes on all substrates, a small number of cells on uncoated EGC did not express K14 (Fig. 6A). While the expression of K10 was not observed in keratinocytes on any substrate, involucrin expression was detected in many cells and on all substrates. However, a higher proportion of keratinocytes were positive for involucrin when grown on uncoated EGC, as compared to keratinocytes on Fib-Mat or collagen I (Fig. 6A).

Keratinocytes on each of the substrates were also examined for p63 expression, a marker associated with a lack of differentiation in keratinocytes. As shown in Fig. 6B, while p63 expression was detected in keratinocytes on all substrates, the number of cells expressing p63 was significantly (p < 0.001) higher on Fib-Mat (93.98%) and collagen I (95.11%) than on TCP (85.48%) (Fig. 6Bii).

Keratinocytes grown on Fib-Mat appeared to retain a smaller size than keratinocytes similarly cultured on collagen I or TCP (Fig. 5A). An assay based on the Haase *et al*.^25^ study was used to analyse the size of individual keratinocytes and this revealed a statistically significant difference (p ≤ 0.05) in the size of keratinocytes grown on Fib-Mat or collagen I in all three replicate experiments (Fig. 5B). The majority of keratinocytes on Fib-Mat were small cells, whilst collagen I had the greatest number of large flat keratinocytes, a characteristic associated with terminal differentiation. While there were differences in size between cells on Fib-Mat and TCP, the differences were not statistically significant.

**Figure 7:**
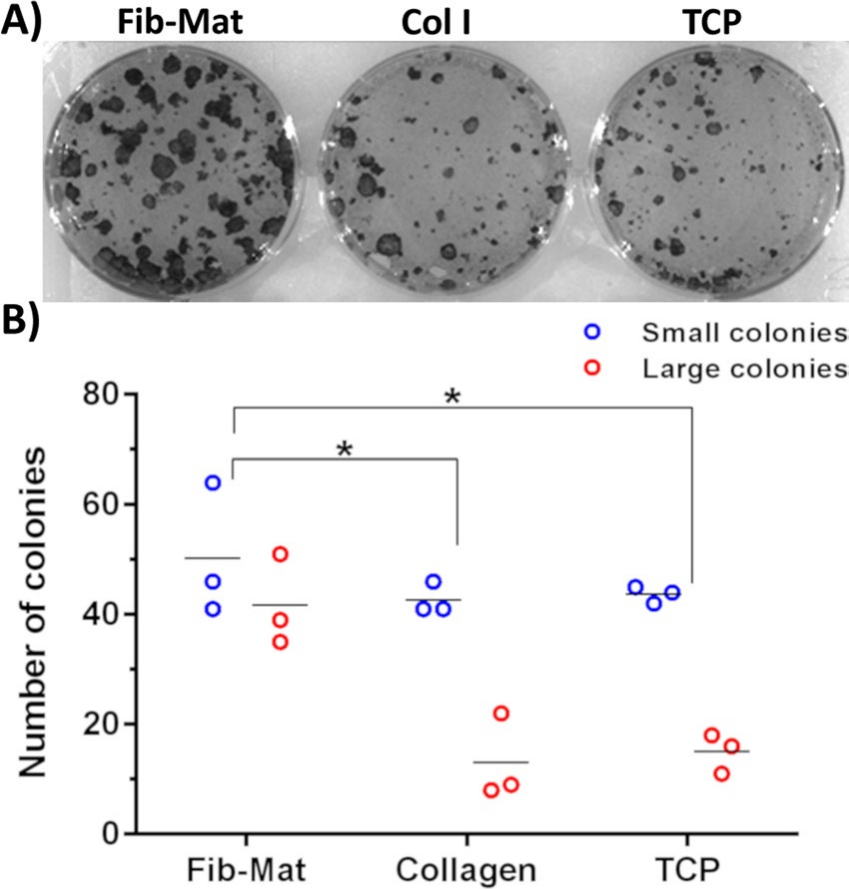
Colony forming ability of keratinocytes previously cultured on different substrates. (**A**) Representative image of the colonies formed by keratinocytes previously cultured on different substrates. Keratinocytes were cultured for three days on Fib-Mat from ATCC PCS-201-012, collagen I or uncoated TCP, the cells were harvested and single cells were seeded at low density onto mitomycin C-treated 3T3-J2 feeder cells, with 3 replicate wells for keratinocytes from each substrate. After 12 days keratinocyte colonies were stained with toluidine blue and imaged. Replicate experiments performed with Fib-Mat from EBL028 and collagen I produced very similar data. (**B**) Quantification of the colonies. All colonies were counted and categorised: large colonies were ≥ 1mm^2^, and small colonies were cell clusters <1 mm^2^. The data from 3 replicate wells are shown, and are representative of three separate experiments. *p < 0.01

To evaluate the self-renewal capability of keratinocytes grown on different substrates, their colony forming ability was examined. Keratinocytes grown on either Fib-Mat, collagen I or TCP were harvested, then seeded onto a layer of mitomycin C-treated feeder cells and grown for 12 days. The number of large colonies ( ≥ 1 mm^2^) produced by keratinocytes “conditioned” by prior growth on Fib-Mat was higher than that seen for keratinocytes previously grown on either collagen I or TCP, and overall the number of colonies obtained for the Fib-Mat conditioned cells was significantly different (P < 0.01) from that obtained for the other cells (Fig. 7).

### Keratinocytes are highly motile on dermal fibroblast-derived matrix

Cells were seeded onto EGC or TCP either uncoated, or coated with Fib-Mat or collagen I, and left overnight to adhere before time-lapse images were taken at 15-minute intervals over a 2 day period. Distinct colonies were only seen when keratinocytes were seeded on uncoated EGC or TCP. Pseudo-colonies, wherein keratinocytes migrated as a group to form a colony, but then dispersed or combined with other colonies, were observed for cells on Fib-Mat regardless of the underlying surface. Keratinocytes on collagen I coated EGC also formed pseudo-colonies, whereas on collagen I coated TCP, all keratinocytes migrated as single cells.

Interestingly, the majority of keratinocytes grown on Fib-Mat, but not collagen I or TCP, remained highly motile over the entire 2 day period. Although keratinocytes plated on collagen I were also motile at early time points, a proportion of the keratinocytes became less motile over time, and reduced motility was accompanied by increased cell size. Keratinocytes on uncoated substrates were the least motile, and an increase in cell size was also observed. (Links to time-lapse videos are given in the supplementary information.)

Cell motility is linked to the organization of the actin cytoskeleton. To examine the arrangement of filamentous actin (F-actin) keratinocytes were grown on Fib-Mat, collagen I or EGC for 3 days before being stained with phalloidin-Alexa Fluor 488. On collagen I and EGC, well-developed actin stress fibres were observed, particularly at the keratinocyte circumference. This was more prominent in the large keratinocytes. In contrast, circumferential stress fibres were less visible in keratinocytes on Fib-Mat (Fig. 5Aii).

### Keratinocytes form stable sheets on dermal fibroblast-derived matrix

**Figure 8:**
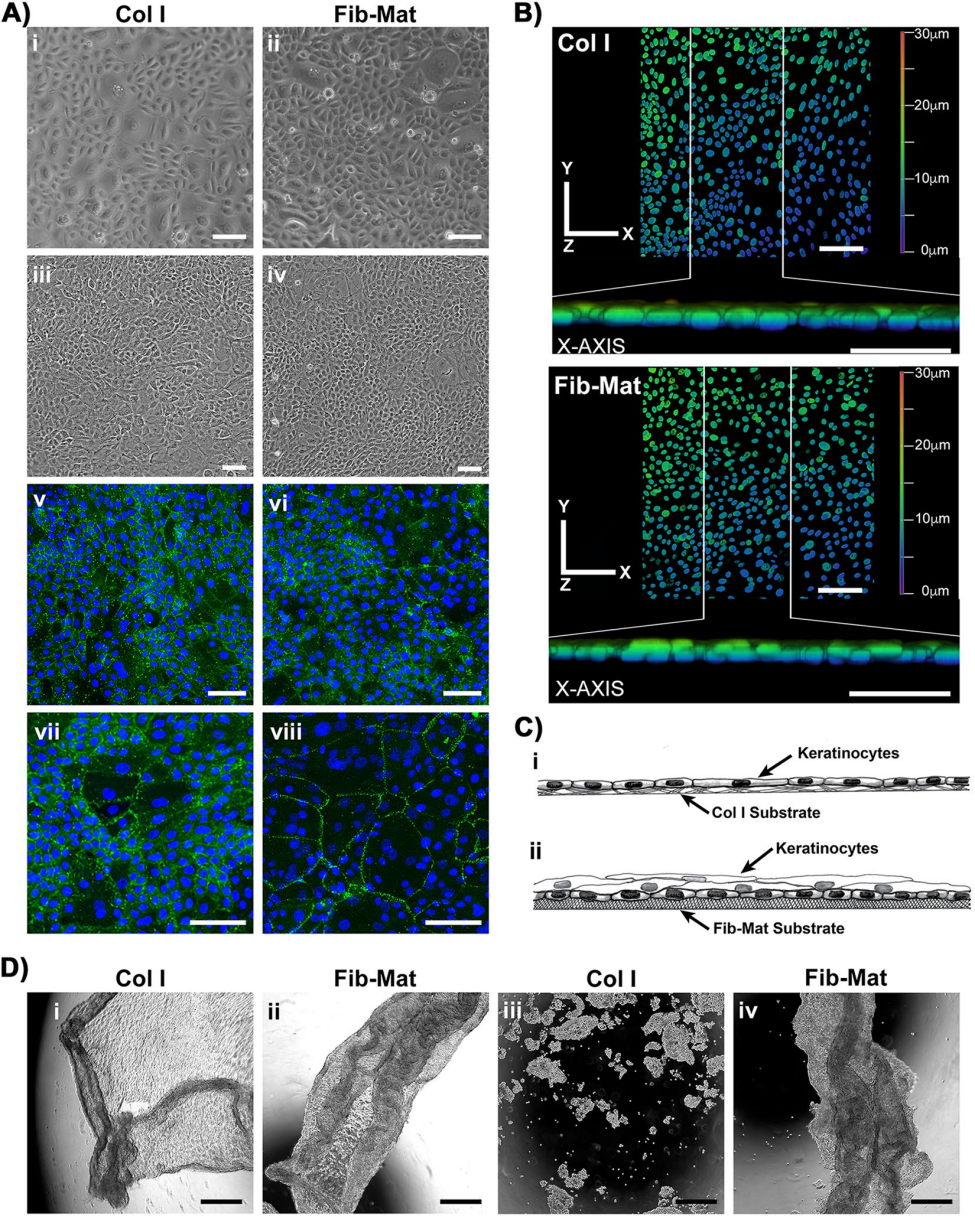
Keratinocyte sheets are more stable when cultured on Fib-Mat (**A**) The morphology of keratinocyte sheets growing on dermal Fib-Mat and collagen I (Col I) substrates. Keratinocyte sheet morphology on day 4 post seeding in DSFKM (i, ii) and on day 5 after 24 hrs in DMEM/Ham’s F12 with 2% human serum (iii, iv) imaged by phase contrast microscopy. Keratinocyte sheets, day 5, stained with an anti-E-cadherin mAb (v-viii) and visualised by confocal microscopy. Shown are overview images indicating the keratinocytes have formed E-cadherin rich cell-cell junctions (v, vi). Higher magnification images revealed keratinocytes formed a monolayer on Col I (vii) but multilayered areas were common on Fib-Mat (viii). Nuclei were stained using DAPI (blue). Scale bars are 100 μm. Representative images of four replicate experiments are shown. (**B**) Keratinocytes sheets cultured on Col I (top) and Fib-Mat (bottom) substrates. Three-dimensional Z-stacked confocal images showing keratinocyte DAPI-stained nuclei as an X-Y projection, and a Z projection along the X-axis of the X-Y image delineated by the white vertical lines. The colour coding indicates the depth of the nuclei within the cell layer. Scale bars are 100 μm. (**C**) Schematics of our interpretation of the data in (**A,B**) of this figure: Col I (i), Fib-Mat (ii). (**D**) Keratinocyte sheets dissociated from the Col I and Fib-Mat substrates after treatment with dispase, and imaged by phase contrast microscopy (i, ii). Appearance of keratinocyte sheets after being subjected to 60 mechanical inversion cycles (1 cycle/sec) on a rocker, as captured by phase contrast microscopy (iii, iv). Scale bars are 500 μm. Data were consistent across three replicate experiments.

Keratinocytes grown to confluence in DKSFM on either collagen I or Fib-Mat were placed in DMEM/Ham’s F12 medium containing human serum for 24 h to encourage the formation of cell-cell junctions. Prior to the medium change, differences in the frequency of large flat cells were apparent in the two cultures, with more areas of small, cobblestone-like keratinocytes visible on Fib-Mat (Fig. 8Ai,ii). After 24 h in the higher calcium containing medium the keratinocytes appeared to tighten both their cell-cell junctions and their adhesion to the substrate, giving the appearance of very flat cell sheets regardless of substrate (Fig. 8Aiii,iv). E-cadherin-rich cell-cell junctions were visible throughout both cultures (Fig. 8Av,vi). The higher magnification images of keratinocytes on collagen I (Fig. 8vii) revealed that all areas encircled by E-cadherin stained junctions contained only one nucleus regardless of the size of the encircled area. In contrast, on Fib-Mat E-cadherin staining encircled both areas with one nucleus and larger areas with multiple nuclei, the latter were readily visible at the higher magnification (Fig. 8Avi,viii). Three-dimensional Z-stacked confocal images of these cultures indicated a monolayer on collagen I, whereas on Fib-Mat at least two layers of nuclei were seen (Fig. 8B). Collectively these data are consistent with the following interpretation. Keratinocytes on the collagen substrate remained as a single layer regardless of their stage of differentiation. However, on Fib-Mat, they formed multiple layers, with the large cells (squames) being uppermost and the DAPI stained nuclei from the small cells, which were attached to the substrate, being visible through the upper cell layer. This is illustrated in the schematic (Fig. 8Ci,ii).

To investigate the stability of the keratinocyte cell-cell junctions the cells were dissociated from their substrates using dispase. The keratinocytes lifted off both collagen I and Fib-Mat as a contiguous cell sheet under these conditions (Fig. 8Di,ii). However, cell sheets lifted from collagen I substrates were markedly more fragile than the cell sheets from Fib-Mat, the former disintegrated into clumps of cells after only 15 mechanical inversion cycles, whereas the latter remained intact after 60 mechanical inversions indicating these sheets were very robust (Fig. 8Diii, iv). These findings were consistent across three replicate experiments.

## Discussion

The regenerative ability of keratinocyte stem cells has been known since the 1980s and has been well described in numerous studies^26–30^. However, the expansion of keratinocytes *in vitro* for clinical use has remained challenging, particularly as the use of undefined xenogeneic materials in the treatment of patients runs counter to best practice for cell therapy, due to concerns about xenopathogens^31^. Despite this, progress towards producing fully functional keratinocytes from feeder-free cultures has been limited. Currently, keratinocytes can be expanded *in vitro* using a defined serum-free medium, and a collagen I matrix to support cell attachment and growth^6,7,32^. However, the prolonged culture of keratinocytes in this regime induces phenotypic changes, especially a diminished capacity for self-renewal and an increased commitment towards differentiation or senescence^7,33,34^. Together, these changes limit the usefulness of cells expanded in this manner for clinical applications. A critical factor for the long-term expansion of keratinocyte stem cells is their natural microenvironmental niche^8^ and a key component of this niche is a native ECM, which is lacking if cells are grown on collagen I or TCP in a serum-free culture system. To recapitulate the dermal niche of keratinocytes *in vivo*, we developed a method to generate a xenogeneic-free dermal fibroblast matrix (Fib-Mat) to use as a substrate for keratinocyte expansion *in vitro*. This Fib-Mat substrate used with defined serum-free medium is shown here to better support and sustain proliferation of undifferentiated keratinocytes than substrates of collagen I or TCP. In addition, keratinocytes expanded on Fib-Mat can be induced to form stable, multilayered keratinocyte sheets that remain intact even after considerable mechanical stress.

The production and use of cell-derived matrices to support the proliferation of undifferentiated stem cells is not new^13,16,35–38^. Similarly, MMC has previously been demonstrated to facilitate keratinocyte stratification and differentiation^21^. However, the generation of xenogeneic-free Fib-Mat using a tissue culture process that includes MMC during the deposition of the ECM, combined with PLA_2_ for decellularisation, is novel. The inclusion of MMC reagents in the primary dermal fibroblast xenogeneic-free cultures was found, robustly and reproducibly, to generate extracellular matrices that completely covered the surface of the culture plates. The PLA_2_ decellularisation protocol generated acellular matrices that were free of cell and nuclear components. The decellularisation protocol appeared to have minimal impact on the ECM content and ultrastructure, and the proteomics analysis indicated that the Fib-Mat has a core matrisome protein composition that is similar to that reported for the dermis *in vivo*^39,40^ and the ECM composition from both donors were very similar. Interestingly, compared to proteomic data derived from skin dermis, the Fib-Mat appeared to lack many of the matrisome-associated proteins. As some of the matrisome-associated proteins are cell-associated proteins (e.g. LGALS1, LGALS3 & GPC1) present in a full skin biopsy, they would be absent in the decellularised Fib-Mat. Furthermore, as Fib-Mat is the result of a monolayer culture of dermal fibroblasts, ECM proteins contributed by other cells resident in the skin (e.g. keratinocytes & melanocytes) will also be absent. The data shown here is predominately using Fib-Mats from ATCC PCS-201-012, but replicate experiments using Fib-Mats from EBL028 gave very similar data. Therefore, we are confident that our observations are not donor cell specific.

Significantly more keratinocytes adhered to Fib-Mat than to collagen I or TCP (Fig. 3B), and possibly because of this, greater keratinocyte proliferation (as determined by cell number and Ki67 expression) was seen on Fib-Mat than on the other substrates (Fig. 3C,D). These data agree with other studies using stem cells in other tissue niches, which reported that cell-derived matrices matching the tissue microenvironment of the cells *in vivo* better supported and sustained the attachment and proliferation of mesenchymal stem cells^41–43^ and synovium-derived stem cells^44,45^. Whether the proliferation we observed can be further increased without compromising the regenerative potential of the system remains to be seen. For example, it will be of interest to determine the nature and cause of the initial lag period before exponential growth occurred on Fib-Mats (Fig. 3C). The primary keratinocyte populations used in this study were neonatal in origin and it will be important to confirm these proliferation data with adult keratinocyte populations from a number of different donors. However, our preliminary observations of the response of adult keratinocytes to the dermal matrix suggest they behave similarly to the neonatal cells, but these data were qualitative (data not shown).

Stronger adhesion may underlie the repression of terminal differentiation seen in cells grown on Fib-Mat and revealed by antibody staining for markers of differentiation, from keratins to involucrin. The basal cell marker keratin K14 was expressed by keratinocytes grown on all three substrates, although there were a small number of cells cultured on EGC which lacked K14 staining. Loss, or down regulation, of K14 could possibly have occurred as a precursor to differentiation^46^. No K10 expression (seen in suprabasal, differentiating keratinocytes) was detected in cells expanded on any of the three substrates. Involucrin, however, was expressed by keratinocytes grown on all substrates, with a higher proportion of positive keratinocytes present on uncoated EGC. Thus, sporadic loss of K14, an increase in involucrin, and a decrease in proliferation were all seen in keratinocytes grown on TCP and EGC, but not on Fib-Mat. These data suggest that keratinocytes on TCP/EGC are more prone to initiating differentiation in the absence of stratification than keratinocytes on Fib-Mat. However, K14 expression was not tightly associated with cell proliferation rates because there was no apparent decrease in K14 expression, despite reduced keratinocyte proliferation, on collagen I.

The pattern of K16 expression was potentially informative, as fewer keratinocytes grown on Fib-Mat expressed K16 than on the TCP and EGC. Together with K6a, K6b and K17, K16 behaves like a stress response protein; it is associated with hyperproliferation and migration during wound healing and is a marker of activated keratinocytes^47,48^. K16 is commonly expressed by suprabasal keratinocytes in hyperproliferative conditions such as psoriasis, squamous carcinoma and wound healing, as well as in most tissue culture systems. From our data it is clear that K16 expression is not correlated with proliferation. The low level of K16 expression by keratinocytes on Fib-Mat may indicate that, despite their increased proliferation, these cells are not mimicking a wound healing response, and keratinocytes on Fib-Mat may also be less stressed than most keratinocytes in tissue culture.

A functional link between p63 expression and keratinocyte stem cell maintenance has been shown by Mills *et al*.^49^ and Yang *et al*.^50^ using a p63-/- mouse. Further studies by Parsa *et al*.^51^ showed that p63 expression is restricted to keratinocytes with high proliferative potential that reside within the basal layer. They also found that p63 was absent from keratinocytes that were terminally differentiating. Most keratinocytes grown on Fib-Mat or collagen I expressed p63, whereas fewer keratinocytes expressed p63 when cultured directly on TCP (Fig. 6B). Interestingly, reduced p63 expression of keratinocytes on TCP coincided with their decline in growth potential (Fig. 3C) and an increase in involucrin expression (Fig. 6A). This inability of uncoated TCP to support the growth of undifferentiated keratinocytes is consistent with the data of others^6^.

Keratinocytes increase in size as they differentiate, a characteristic that has been observed *in vivo* during normal epithelial maturation and in *in vitro* cultures^7^. The change in keratinocyte size, and particularly the change in the ratio of the areas occupied by the nucleus and the cytoplasm, may indicate keratinocytes undergoing terminal differentiation, as keratinocyte enlargement accompanied by the expression of involucrin has been reported^52,53^. Other studies found that small keratinocytes are undifferentiated and retain a high proliferative capability^54–56^. Hence, numerous investigators have described cell size as a criterion distinguishing keratinocyte stem cells from keratinocytes committed towards differentiation^8,55,56^. We found more small keratinocytes were present in cultures grown on Fib-Mat, whereas culturing the same keratinocyte population on collagen I produced larger cells (Fig. 5). These data are consistent with Esteban-Vives *et al*.^7^.

Recently Nanba *et al*.^57^ suggested that cell motility is an attribute of undifferentiated keratinocytes. They reported that highly motile keratinocytes and keratinocyte colonies were indicative of undifferentiated keratinocytes with high proliferative capabilities. We found keratinocytes grown on Fib-Mat were very motile (See video in supplementary information). Tight keratinocyte colonies formed only on TCP, whereas looser pseudo-colonies formed on Fib-Mat and collagen I. In addition, most keratinocytes grown on Fib-Mat were highly motile throughout the experiment, but keratinocytes cultured on collagen I and TCP were less motile, a trait very evident when the time in culture was extended. Actin filament organisation differed between the cultures as predicted by their different motility. Keratinocytes plated on collagen I or EGC without a matrix protein coating developed a circumferential actin network (Fig. 5Aii), similar to that reported by Nanba *et al*.^58^ and which was described to be indicative of reduced cell movement and terminal differentiation. In contrast, keratinocytes grown on Fib-Mat had short bundles of actin that were radially distributed (Fig. 5Aii), an arrangement of actin filaments described as indicative of proliferative, undifferentiated keratinocytes^58^. Moreover, the reduced motility of individual keratinocytes was accompanied by an increase in cell size. The association of increased size and decreased motility during keratinocyte differentiation was reported many years ago by Sun and Green^52^ and our results are consistent with these findings.

Collectively our data indicate that neonatal keratinocytes grown on Fib-Mat are less differentiated than the same keratinocytes cultured on TCP, which show signs of undergoing early commitment to terminal differentiation. Despite exhibiting larger cell sizes, lower cell motility and lower colony forming ability characteristics that suggest early terminal differentiation, keratinocytes grown on collagen I still expressed markers (K14 and p63) of undifferentiated keratinocytes. Others have also reported that keratinocytes can retain some markers characteristic of undifferentiated cells during the early stages of differentiation. Webb *et al*.^59^ found that keratinocytes in the basal layer of the epidermis do not switch off the expression of keratin 15 (a marker of keratinocyte quiescence, and in some circumstances of stem cells) even during the differentiation process. Esteban-Vives *et al*.^7^ also observed that keratinocytes grown on collagen I retained K15 expression despite showing signs of differentiation. Hence, it is likely that on collagen I, keratinocytes are in the early stages of terminal differentiation, even though some markers of undifferentiated cells are present.

This conclusion was further supported by the behaviour of the keratinocytes expanded on the different substrates in a colony forming assay. Barrandon *et al*.^60^ described the use of colony forming assays as an invaluable tool for determining the presence of stem cells within a primary, freshly isolated keratinocyte population. Undifferentiated keratinocyte stem cells have a higher self-renewal capability and therefore can form large, progressively growing colonies (>1 mm;^2^ holoclones). We found that the primary, neonatal keratinocytes that we used when expanded on Fib-Mat produced more large colonies than keratinocytes expanded on the other two substrates. This indicated that Fib-Mat “conditioning” better retained and promoted the self-renewal ability of keratinocytes compared to keratinocytes from the other substrates (Fig. 7).

Others have also shown cell-ECM interactions are important for preserving the self-renewal ability of cultured keratinocytes. Adams and Watt^61^ demonstrated that keratinocytes that lose ECM contact are triggered to terminal differentiation. Similarly, we show here that cells adhered better to Fib-Mat and also showed resistance to terminal differentiation. Likewise, our data and that of Coolen *et al*.^6^ show that keratinocytes underwent terminal differentiation when grown on tissue culture plastic that lacked an ECM protein. Hence, ECM proteins such as collagen I^7^, collagen IV^6^ and fibronectin^62^ have been used as substrates to culture keratinocytes. Although using these single ECM proteins enable the keratinocytes to adhere and proliferate, they do not sustain the long-term growth of keratinocytes^7,34^. In this reductionist approach, the synergistic impact of growth factors and ECM proteins and their coordinated signalling pathways in the keratinocytes is overlooked. Others have shown that the combination of three matrix proteins can have a synergistic effect^63–65^. Flaim *et al*.^63^ found that the combination of collagen I with laminin and collagen III enabled embryonic stem (ES) cells to efficiently differentiate towards a liver progenitor lineage, although individually these matrix proteins were unable to promote liver progenitor cell differentiation. Furthermore, Watt *et al*.^62^ showed that substrates comprising a combination of laminin, collagen IV and fibronectin inhibited the differentiation of keratinocytes during *in vitro* culture.

The proteomics data indicated our Fib-Mat contained laminin, collagen IV and fibronectin plus numerous other ECM proteins, and some ECM associated proteins, but very few of the secreted factors found in the dermis. Our data indicate the combined signals of the core matrisome proteins and the ECM associated proteins that are present in Fib-Mat, are sufficient to suppress keratinocyte differentiation and to promote proliferation. Essential growth factors for keratinocyte proliferation were probably present in the culture medium, and the Fib-Mat provided the ECM components to correctly present these growth factors to the growing keratinocytes.

The defined keratinocyte serum free medium used in this study has a low calcium concentration and was developed to promote keratinocyte monolayer formation without stratification, whereas elevated calcium concentrations, in association with other signals, increase the rate of differentiation and promote and stabilise cell-cell adhesion and stratification^66,67^. Accordingly, to investigate keratinocyte sheet formation, at cell confluence the DKSFM was replaced with the medium traditionally used to culture keratinocytes on murine fibroblast feeder layers, but supplemented with human serum in accordance with the xenogeneic-free culture goals of this study. The keratinocyte morphology changes observed 24 h following the medium change mirrored those reported previously for keratinocytes grown on a collagen substrate where calcium was added to the serum free medium^68^. However, in the published report keratinocytes were grown to confluence from seeding in either the low or high calcium media. In both cases cells in the high calcium medium were flatter and greater inter-cell adhesion was observed, possibly due to the calcium concentration facilitating desmosomal connections to the underlying substrate, and inter-cell junction formation^69^. In our study the contribution of E-cadherin mediated cell-cell adhesion in the high calcium medium was demonstrated for keratinocytes on both substrates. However, the striking difference in the E-cadherin staining pattern that was consistently observed indicated that on Fib-Mat the sheets were multi-layered, whereas on collagen I they were one cell thick. The Z-stack images of the positions of the nuclei within the cell sheets confirmed this interpretation (Fig. 8A,B). Thus, on Fib-Mat, when confluent and in medium with an appropriate calcium concentration the keratinocytes differentiated forming multiple layers with the squames uppermost. Possibly these squames expressed involucrin whereas the cells remaining attached to the matrix were undifferentiated and lacked involucrin expression. Such a pattern of expression was observed in our preliminary experiments in which keratinocytes were cultured from seeding on Fib-Mat in the high calcium medium (data not shown). Others reported that keratinocytes continuously expanded in serum free keratinocyte medium failed to stratify correctly when in a skin-equivalent culture using decellularised human dermis, and that the addition of calcium and serum to the serum free medium were required to trigger stratification and terminal differentiation over a 14 day period^69^.

The other difference in the keratinocyte sheets formed on the two substrates was their strength and stability. The sheets from collagen I substrates readily disassociated, whereas the sheets from Fib-Mat were very stable and remained intact when placed under considerable mechanical stress (Fig. 8D). The multi-layered nature of the latter cell sheets probably contributed to their greater stability over the cell sheets developed on collagen I substrates. These data indicate that viable fibroblasts are not required for stable keratinocyte sheet formation, but an ECM from dermal fibroblasts provides sufficient signals to induce strong inter-cell junctions and the formation of a cell sheet two to three cells thick. Hence, the keratinocyte sheets that form on the Fib-Mat resemble those which are developed using a monolayer of J2–3T3 cells and medium containing FBS^70^, but our system is free of xenogeneic materials and the cell sheets form quite rapidly. Nevertheless, further work is required to determine whether these cell sheets can be successfully used clinically for wound healing applications.

In conclusion, this study highlights the role of an acellular dermal fibroblast ECM in modulating keratinocyte growth and differentiation. We describe a novel culture system, using acellular dermal fibroblast ECM, which was produced using MMC and PLA_2_. This is an improvement on the current protocol for the defined keratinocyte serum-free culture system used in this study, as using the acellular matrix as a substrate allows amplification of undifferentiated neonatal keratinocytes that retain many features of stem/progenitor cells. In contrast, neonatal keratinocytes expanded using defined serum free medium and collagen I (the protocol recommended by the manufacturer of the medium) are likely to have progressed down the terminal differentiation pathway as a result of the expansion protocol used. In addition, keratinocytes grown to confluence on the acellular matrix produced multi-layered cell sheets when placed in a higher calcium containing medium. The robust nature of these keratinocyte sheets suggests our protocol is a significant step forward in xenogeneic free culture techniques used to prepare keratinocytes for grafting. However, further validation of this method will require testing the acellular dermal fibroblast ECM with a variety of different keratinocyte populations originating from a number of adult donors, and the testing of the epidermal sheets *in vivo*.

## Materials and Methods

### Antibodies

The primary rabbit polyclonal antibodies used were: anti-collagen I (Abcam; Cambridge, UK), anti-collagen IV (Abcam), anti-fibronectin (Abcam) and the anti-perlecan antibody CCN-1 was a gift from Prof. John Whitelock (University of NSW, Sydney, Australia). The mouse monoclonal antibodies (mAbs) used were: anti-fibroblast marker (clone TE7; Millipore; MA, USA), anti-involucrin (clone SY5; Sigma; MO, USA), anti-ki67 (clone MM1; Novacastra; Wetzlar, Germant), anti-p63 (clone 4A4; Abcam), anti-α-smooth muscle actin (clone 1A4; Sigma), anti-Thy1 (clone 5E10, BD Bioscience; NJ, USA) and anti-vimentin (clone V9; Dako; CA, USA). The mouse mAbs anti-keratin 14 (K14, clone LL001), anti-keratin 16 (K16, clone LL025) and anti-keratin 10 (K10, clone LH2) were produced in the labs of EBL and IM Leigh^71,72^. The secondary antibodies used were Alexa488 anti-mouse IgG, Alexa546 anti-mouse IgG, Alexa 488 anti-rabbit IgG and Alexa546 anti-rabbit IgG (all from Molecular Probes, ThermoFisher Scientific; OR, USA).

### Cell culture

Two sources of primary adult human dermal fibroblasts (HDF) were used: one cell population was obtained from the American Type Culture Collection (ATCC PCS-201-012; VA, USA) and the other (EBL028) from the Skin Cell Bank of the Institute of Medical Biology (IMB), Singapore. The donors were aged 34 and 23 years respectively. The HDFs from the Skin Cell Bank of the IMB were collected in accordance with relevant guidelines and regulations and with fully informed donor consent. Use of these cells was approved by the Institute of Medical Biology (ethics approval no. IRB/2011/418/D) and Curtin University Human Ethics committee (reciprocal approval). All experiments were performed in accordance with relevant guidelines and regulations. HDFs were maintained in Dulbecco’s Modified Eagle’s medium (DMEM) supplemented 10 mM HEPES, 2 mM L-glutamine and 1 mM sodium pyruvate (all from Gibco, ThermoFisher Scientific) and 10% FBS (Serana Europe GmBH; Pessin, Germany). HDF from passage 7 to 15 were used for all experiments. Human neonatal keratinocytes (purchased from Gibco) were cultured on tissue culture growth surfaces that have been coated with collagen I (Sigma) in PBS (3 µg/cm^2^) and maintained in defined keratinocyte serum free medium (DKSFM; Gibco). Keratinocytes at passage 4 or 5 were used for all experiments. Keratinocytes from three different lot batches were used in this study.

### Extracellular matrix deposition with macromolecular crowding treatment

HDFs were seeded at a density of 1.5 × 10^4^ cells/cm^2^ and were allowed to attach overnight in basal medium comprising DMEM: Ham’s F12 (3:1) supplemented with 2% human serum, 10 mM HEPES, 2 mM L-glutamine, 1 mM sodium pyruvate (all from Gibco) and 30 µg/ml ascorbic acid (Wako Chemical; Toyko, Japan). The medium was then replaced with fresh medium containing 7.5 mg/ml Ficoll 70 (Sigma) and 25 mg/ml Ficoll 400 (GE Lifesciences; Buckinghamshire, UK) to induced MMC. The HDFs were cultured for 6 days for ECM deposition, with the medium being changed on alternate days.

### Decellularization of dermal fibroblast-derived extracellular matrix

The ECMs deposited using MMC were decellularised using either EDTA, ammonium hydroxide (AH) or phospholipase A_2_ (PLA_2_). For the ET method, cells were rinsed with PBS and 2.5 mM EDTA/PBS, and then incubated in 2.5 mM EDTA/PBS for 10 min at 37 °C. Using a P1000 pipet, the cell monolayer was dislodged from the culture leaving the matrix behind. The matrix was washed with PBS, incubated for 5 min at 37 °C with 0.5% Triton X-100/PBS and washed with PBS. For AH decellularization, the cells were washed with PBS and incubated in 0.02 M ammonium hydroxide (Sigma)/0.5% Triton X-100/1x EDTA-Free protease inhibitor (Roche; Basel, Switzerland) at 37 °C for 5 min. For the PLA_2_ method, the cells were washed in PBS and incubated in PLA_2_ (20 U/ml) (Sigma)/50 mM Tris-HCl (pH 8)/0.15 M NaCl/1 mM MgCl_2_/1 mM CaCl_2_/0.5% sodium deoxycholate/1x EDTA-Free protease inhibitor (Roche) at 37 °C for 30 min. Matrices decellularised by the AH and PLA_2_ methods were treated with 0.02 mg/ml DNase I (Amresco, PA, USA) in reaction buffer (10 mM Tris-HCl (pH 7)/2.5 mM MgCl_2_/0.5 mM CaCl_2_) at 37 °C for 30 min and then washed with PBS. The presence of DNA in decellularized ECM was determined by staining with 4′,6-diamidino-2-phenylindole (DAPI; Sigma; 1 µg/ml in PBS), while the presence of residual actin in decellularized ECM was determined by staining with 1 unit/ml of Phalloidin conjugated with tetramethylrhodamine (TRITC). Images were captured using a Zeiss LSM510 inverted fluorescent microscope. In addition to visualizing DNA using DAPI, residual nucleic acids in the decellularized ECM were measured using the CyQUANT cell proliferation assay kit (Molecular Probes, Thermo Fisher Scientific) following the manufacturer’s protocol. Fluorescence intensity was measured with 485 nm/535 nm filters using an EnSpire Multimode Plate Reader (Perkin Elmer; MA, USA).

### Immunocytochemistry analysis

Immunofluorescent staining was performed on cells or ECM adhered to etched glass coverslips in 24-well plates. Coverslips were prepared as described in Chaturvedi *et al*.^73^ The cell or ECM layer was fixed with 4% paraformaldehyde in PBS for 15 min at RT, washed with PBS then blocked with 10% goat serum/1% BSA/PBS for 1 h at RT. Blocking solution was removed, and samples were incubated for 1 h at RT with primary antibody prepared in 10% goat serum/1% BSA/PBS. Cells were washed 3 × 5 min with PBS before being incubated for 1 h with secondary antibodies prepared in 10% goat serum/1% BSA/PBS. The ECMs were washed 3 × 5 min with PBS and incubated in DAPI (1 µg/ml in PBS) for 10 min. Coverslips were mounted in Vectashield antifade mounting medium (Vector Laboratories; Peterborough, UK) and sealed with nail varnish. Images were captured with either a Zeiss Axioskop fluorescent microscope (Carl Zeiss, Germany) using Spot Advanced software (Michigan, USA) or a Nikon A1 + Confocal Microscope (Nikon, Tokyo, Japan). All antibodies were titrated to determine their appropriate concentration for the experiment. To generate a 3D representation of the matrix, Z-stacked images of anti-collagen I antibody stained ECM were obtained using a Nikon A1 + confocal microscope and images were merged using the NIS-Elements AR analysis software.

### Keratinocyte proliferation

The proliferation of keratinocytes on the substrates (Fib-Mat, collagen I (3 µg/cm2) and tissue culture plastic (TCP)) was assessed. Keratinocytes were harvested and seeded at a density of 1 × 10^4^ cells/well in a 48-well tissue culture plate (NUNC, ThermoFisher Scientific) and grown for six days. At 24 h intervals keratinocytes were fixed for 5 min with cold acetone:methanol (1:1), washed with PBS and incubated with PBS/1%BSA for 1 h at RT before the nuclei were stained with DAPI (Sigma). Using an Olympus IX-81 high content screening inverted microscope (Olympus; Tokyo, Japan) and a 10x objective, 7 by 11 non-overlapping quadrants were imaged, to produce a 0.5 cm^2^ area image. Nuclei/cell numbers were determined using Fiji-Image J software^74^ and its “Find Object” macro.

### Keratinocyte adhesion to substrates

Cell adhesion assays were performed in 96 well tissue culture plate (NUNC). Keratinocytes were harvested, resuspended in adhesion assay buffer (DMEM), 10 mM HEPES, 2 mM L-glutamine, 1 mM sodium pyruvate, 0.2% BSA (Sigma), 25 μg/ml adenine (Sigma), 0.4 μg/ml hydrocortisone (SOLU-CORTEF, Pfizer; NY, USA), 0.12 IU/ml insulin (Humulin, Lilly; IN, USA), and seeded at a density of 1 × 10^4^ cells/well and left to adhere for 1 h at 37 °C to either decellularized HDF ECM, collagen I (3 µg/cm^2^) or uncoated TCP. Unbound cells were removed by washing with adhesion assay buffer followed by PBS. The plate was placed overnight at −80 °C and then brought up to RT before the cell number was determined using the CyQUANT cell proliferation assay kit (Molecular Probes, Invitrogen), following the manufacturer’s instruction. Fluorescence intensity was measured with 485 nm/535 nm filter using an EnSpire Multimode Plate Reader (Perkin Elmer). Results were calculated as a percent of the control, which was prepared by pelleting 1 × 10^4^ keratinocytes, washing and storing the pellet at −80 °C before using the CyQUANT assay kit to determine the fluorescence intensity of this number of keratinocytes.

### Keratinocyte size and motility

Keratinocytes were harvested and seeded (1 × 10^4^ cells/well) on the various substrates in the wells of a 48-well culture plate (NUNC). After three days of culture in DKSFM keratinocytes were fixed with 4% paraformaldehyde/PBS for 15 min at RT, incubated in PBS/1%BSA for 1 h at RT before polymerized actin was stained with 1 unit/ml of phalloidin-Alexa 488 (Molecular Probe) and nuclei with DAPI. Using a 20x objective and an Olympus IX-81 high content screening inverted microscope (Olympus), 8 by 8 non-overlapping quadrants were imaged. Cell size as delineated by the stained actin cytoskeleton was determined using the Cell Profiler software^75^.

Keratinocyte movement was assessed on either Fib-Mat, collagen I coating or uncoated TCP. Keratinocytes were seeded as described and following an overnight incubation to allow cell adhesion; live cell images were collected using Olympus IX-81 high content screening inverted microscope (Olympus). Time-lapse images were taken at 15-minute intervals over 2 days using a 10x objective.

### Quantifying Ki67-Positive Keratinocytes

Briefly, keratinocytes were harvested and then seeded as described above and at day 3, keratinocytes were fixed with 4% paraformaldehyde for 15 min at RT, permeabilized with cold 0.1% Triton X-100/PBS for 3 min and then incubated in PBS/1% BSA for 1 h at RT. The keratinocytes were blocked with 10% goat serum/1% BSA/PBS for 1 h at RT before being incubated with a 0.3 µg/ml anti-Ki67 antibody (Novacastra). This was followed by a 1 h incubation with Alexa 488 conjugated anti-mouse antibody at RT. Keratinocyte nuclei were stained using DAPI. Images were taken on the Olympus IX-81 high content screening inverted microscope. Using a 10x objective, 7 by 11 non-overlapping quadrants were imaged. The percent of keratinocytes positive for Ki67 was determined using the Cell Profiler software^75^.

### Quantifying p63-Positive Keratinocytes

Keratinocytes were seeded onto the different substrates, in quadruplicate, in a 24 well culture plate and cultured for 3 days after which they were fixed, permeabilised and blocked as described for the Ki67 expression experiment. Keratinocytes were then incubated with 0.5 µg/ml of anti-p63 mAb and the anti-mouse Alexa 546 conjugated antibody, washed and kept in PBS. A Nikon A1 + confocal microscope and a 10x objective lens were used to image 3 by 3 non-overlapping quadrants per well; with 4 wells per substrate analysed. The percentage of p63 positive keratinocytes was determined using Cell Profiler software^75^.

### Colony forming efficiency assay

Keratinocytes were grown on acellular HDF-derived ECM, collagen I coating or uncoated TCP for 3 days in DKSFM, before being harvested. Keratinocytes (1 × 10^3^) from each substrate were seeded into three replicate wells of 6-well tissue culture plates containing mitomycin C-treated 3T3-J2 feeder cells (courtesy of Yann Barrandon) and cultured for 10–12 days in culture medium which was a 3:1 ratio of Dulbecco’s modified Eagle’s medium and Ham’s F12 supplemented with 10 mM HEPES, 2 mM L-glutamine, 1 mM sodium pyruvate, 25 μg/ml adenine, 0.4 μg/ml hydrocortisone, 0.12 IU/ml insulin, 2 nM triiodothyronine (Sigma), 10 ng/ml epidermal growth factor (BD Bioscience), 5 mM forskolin (Sigma)(DMEM/Ham’s F12) plus 10% FBS. Medium changes were performed on alternate days. After 10–12 days the cells were fixed with (1:1) acetone: methanol and stained with 0.1% toluidine blue in double-distilled water. The colonies that formed were counted, with colonies ≥1 mm^2^ being “large” and the rest “small”.

### Keratinocyte Sheet Disassociation Assay

Acellular HDF-derived ECM and collagen I substrates were prepared. Keratinocytes were harvested and seeded on these substrates at a density of 1 × 10^4^ cells/well in a 24-well tissue culture plate (NUNC) and grown for four days in DKSFM with medium changes on alternate days. After 4 days, the culture medium was replaced with DMEM/Ham’s F12 supplemented with 2% human serum (Sigma), and the cells cultured for an additional 24 hrs. Keratinocyte sheets were fixed, permeabilised with 0.1% Triton-X100 in PBS, blocked with BSA/1% goat serum/PBS, and stained with 20 μg/ml of anti-E Cadherin mAb, 4A2C7 (Zymed, CA, USA), anti-mouse Alexa 488 conjugated antibody and DAPI, as detailed in immunocytochemistry analysis. The keratinocyte sheets were detached from their substrates by incubating at 37 °C for 30 min with 2.4 U/ml dispase (Roche Diagnostics, Basel, Switzerland) in DMEM/Ham’s F12 supplemented with 10 mM HEPES (Gibco). Detached sheets were washed with DMEM/Ham’s F12/HEPES and transferred to a 10 ml tube containing DMEM/Ham’s F12/HEPES. To assess the strength of the keratinocyte sheets, the 10 ml tubes underwent 60 inversion cycles (one inversion cycle/sec) on a rocker before being transferred to a 24-well plate for imaging. Keratinocyte sheets were imaged using a Zeiss LSM510 inverted microscope.

### Mass spectrometry and proteomics analysis

Dermal fibroblast-derived ECMs were generated using MMC and decellularized using PLA_2_. To solubilize the acellular ECM, 8 M Urea/50 mM Tris-HCl pH 8.0 was added before scraping the matrix off the surface and transferring it to a microtube. The matrix mixture was reduced with 10 mM DTT (Sigma), alkylated with 55 mM Iodoacetamide (Sigma) and then diluted with 100 mM TEAB buffer to reach a Urea concentration of < 1 M. The matrix proteins were digested with sequencing grade endoproteinase Lys-C (Promega, WI, USA) and sequencing grade-modified trypsin (Promega) at a ratio of 1:100 at 25 °C for 4 h and 18 h respectively, samples were subsequently acidified with 1% TFA (trifluoroacetic acid) and desalted. Following desalting with a Sep-Pak C18 column cartridge (Waters, Milford MA), the samples were analysed using an Easy nLC 1000 liquid chromatography system (Thermo Fisher Scientific) coupled to an Orbitrap Fusion Mass Spectrometer (Thermo Fisher Scientific). Each sample was analyzed in a 60 min gradient using an Easy Spray Reverse Phase Column (50 cm × 75 µm internal diameter, C-18, 2 µm particles, Thermo Fisher Scientific). Data were acquired in −3 s cycle with the following parameters: MS in Orbitrap and MS/MS in ion trap with ion targets and resolutions (OT-MS 4 × E5 ions, resolution 120 K, IT-MS/MS 1000 ions/turbo scan, “Universal Method”).

### Proteomics Data analysis

The peak list was generated using Proteome Discoverer (Version 1.4. Thermo Fisher Scientific). The MS/MS spectra were searched with the Mascot 2.5.1 (Matrix Science, MA, USA) search algorithm using the Human UniProt Database. The following parameters were used: precursor mass tolerance (MS) 20 ppm, IT-MS/MS 0.6 Da, 3 missed cleavages; Variable modifications: Oxidation (M), Deamidated (NQ), Acetyl N-terminal protein, Static modifications: Carboamidomethyl (C). Forward/decoy search was used for false discovery rate (FDR) estimations on peptide/PSM level, and were set at high confidence, FDR 1% and medium confidence, FDR 5%. The generated protein list was curated using the Matrisome^24^ database.

### Statistical analyses

Statistical analyses were performed using Graph Pad Prism V6 software (GraphPad Software, San Diego, CA). Generally a Kruskal-Wallis test with Dunns multiple comparison test was conducted to take into account the non-normal distribution of the data, and or the small size of the data set. If the data were from multiple separate experiments each experiment was analysed separately and the significance indicated when it was consistent across all experiments. A p-value of p < 0.05 was considered statistically significant. The colony forming assay data required a two-way ANOVA with multiple comparisons.

## Supplementary information

Supplementary Information

Fib-Mat on ECG

Fib-Mat on TCP

Type I Collagen on EGC

Type I Collagen on TCP

Uncoated EGC

Uncoated TCP
