# Supplementary material for: In Vitro Expansion of Keratinocytes on Human Dermal Fibroblast-Derived Matrix Retains Their Stem-Like Characteristics
Source: Sci Rep. 2019 Dec 6;9:18561. doi: 10.1038/s41598-019-54793-9 (PMC6897920; doi:10.1038/s41598-019-54793-9)
Supplement: Supplementary file 1 — Supplementary Information [file 41598_2019_54793_MOESM1_ESM.docx]

**In Vitro Expansion of Keratinocytes on Human Dermal Fibroblast-Derived Matrix Retains Their Stem-Like Characteristics**

**Chee-Wai Wong**^1,2^, Catherine F. LeGrand^1,2^, Beverley F. Kinnear^1,2^, Radoslaw M. Sobota^3^, Rajkumar Ramalingam^4^, Danielle E. Dye^,2^, Michael Raghunath^5^, E. Birgitte Lane^4^, and Deirdre R. Coombe^1,2,6*^

^1^School of Pharmacy and Biomedical Sciences, Faculty of Health Sciences, Curtin University, Bentley, WA 6102, Australia;

^2^Curtin Health Innovation Research Institute, Faculty of Health Science, Curtin University, Bentley, WA 6102, Australia;

^3^Institute of Molecular and Cell Biology, Agency for Science, Technology and Research (A*STAR), 61 Biopolis Drive, No. 07-48A Proteos, Singapore 138673, Singapore;

^4^Skin Research Institute of Singapore and Institute of Medical Biology, Agency for Science, Technology and Research (A*STAR), 8A Biomedical Grove, 06-06 Immunos, Singapore 138648, Singapore;

^5^Centre for Cell Biology and Tissue Engineering, Competence Centre for Tissue Engineering and Substance Testing (TEDD), Institute for Chemistry and Biotechnology, ZHAW School of Life Science and Facility Management, Zurich University of Applied Science, Switzerland

^6^Centre for Cell Therapy and Regenerative Medicine, School of Biomedical Sciences, The University of Western Australia, Crawley, WA

*Corresponding Author: D.Coombe@exchange.curtin.edu.au

**
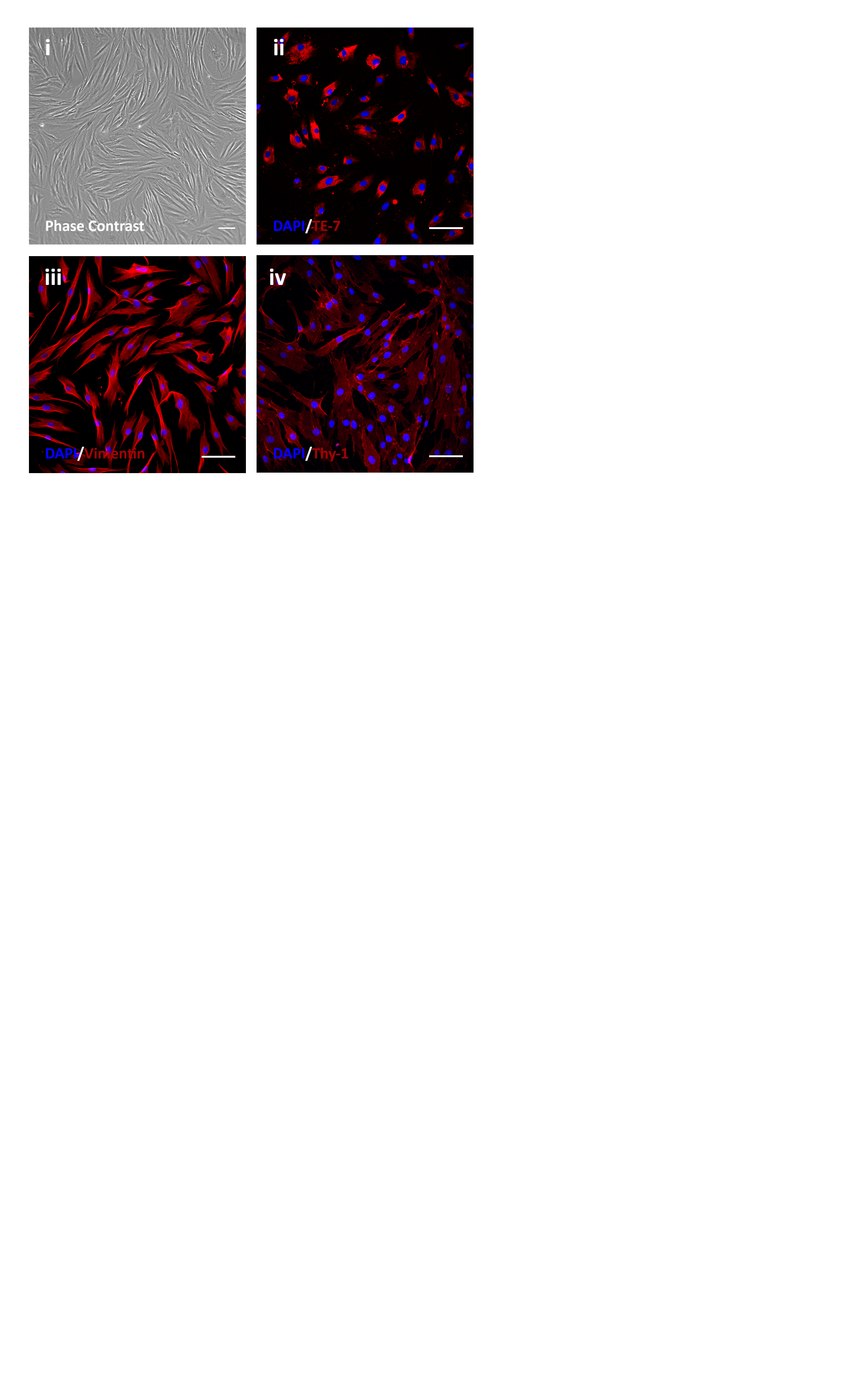
**

**Figure S1: Characterization of human dermal fibroblasts (HDF).**

HDF were stained with antibodies recognising the fibroblast markers: TE-7 (ii), vimentin (iii) or Thy-1 (iv). The secondary antibody was an Alexa Fluor 546-conjugated anti-mouse IgG1. Nuclei were stained with DAPI (blue). Scale bars are 100 µm.

**
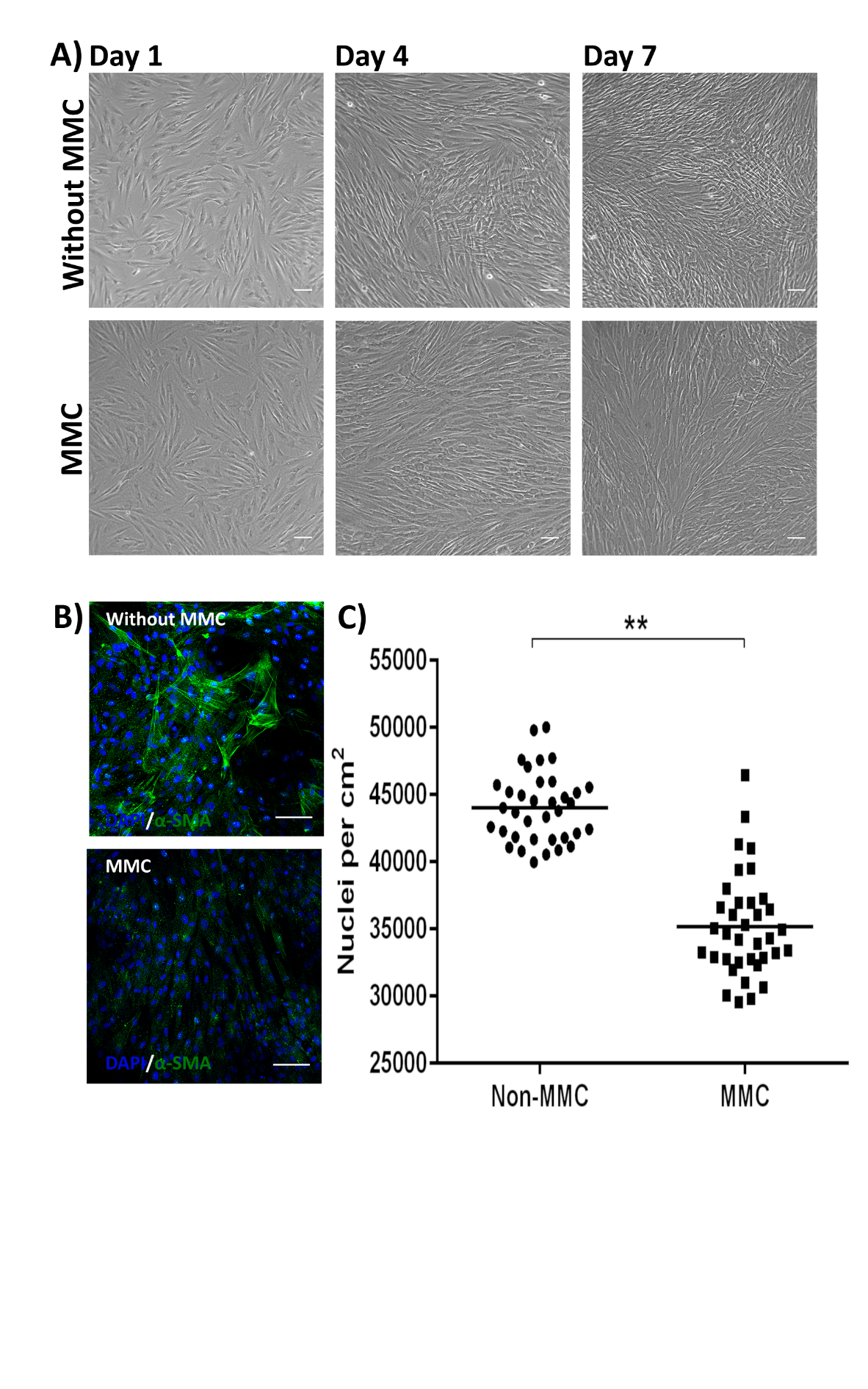
**

**Figure S2: The effect of MMC on fibroblast behaviour.**

**A)** Phase contrast images of HDF cultured with or without MMC. Scale bars are 100 μm.

**B)** Myofibroblast differentiation with and without MMC. HDF were grown either with or without MMC for seven days. The cells were stained with a mAb recognising α-smooth muscle actin (α-SMA) and imaged by confocal microscopy. The secondary antibody was an Alexa Fluor 488-conjugated anti-mouse IgG2a. Nuclei were stained using DAPI (Blue). Scale bars are 100 µm.

**C)** HDF grow more slowly under MMC conditions. The number of HDFs after culturing with or without MMC for seven days. Mean values are given. Shown is a representative of triplicate experiments. ** = p<0.01.

**
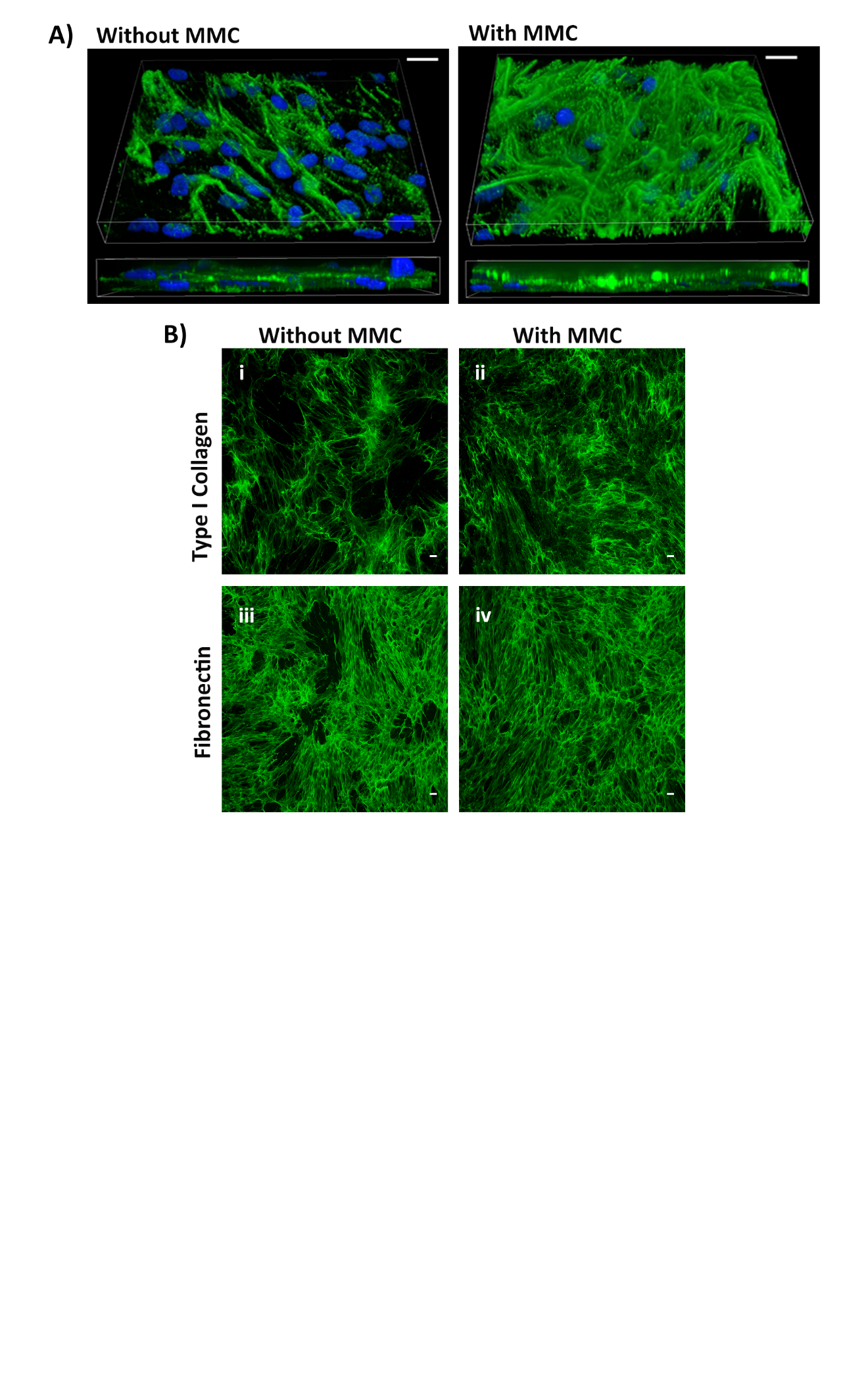
**

**Figure S3: The effect of MMC on the ECM deposited by HDF**

**A)** The effect of MMC on the 3D architecture of the ECM deposited by HDF. A 3D Z-stacked confocal image of type I collagen deposited by HDFs grown without or with MMC for seven days. Cells and matrix were immunostained for type I collagen. The secondary antibody was an anti-rabbit IgG Alexa Fluor 488-conjugated antibody. Nuclei were stained with DAPI (Blue). Scale bars are 10 μm.

**B)** The ECM deposited by HDF with or without MMC after decellularisation. The HDF were grown with or without MMC for seven days. The cell layers were decellularised using PLA_2_. Matrices were immunostained for type I collagen (i, ii) or fibronectin (iii, iv). The secondary antibody was an Alexa Fluor 488-conjugated anti-rabbit IgG. Nuclei were stained with DAPI (Blue). Scale bars are 100 μm.

**
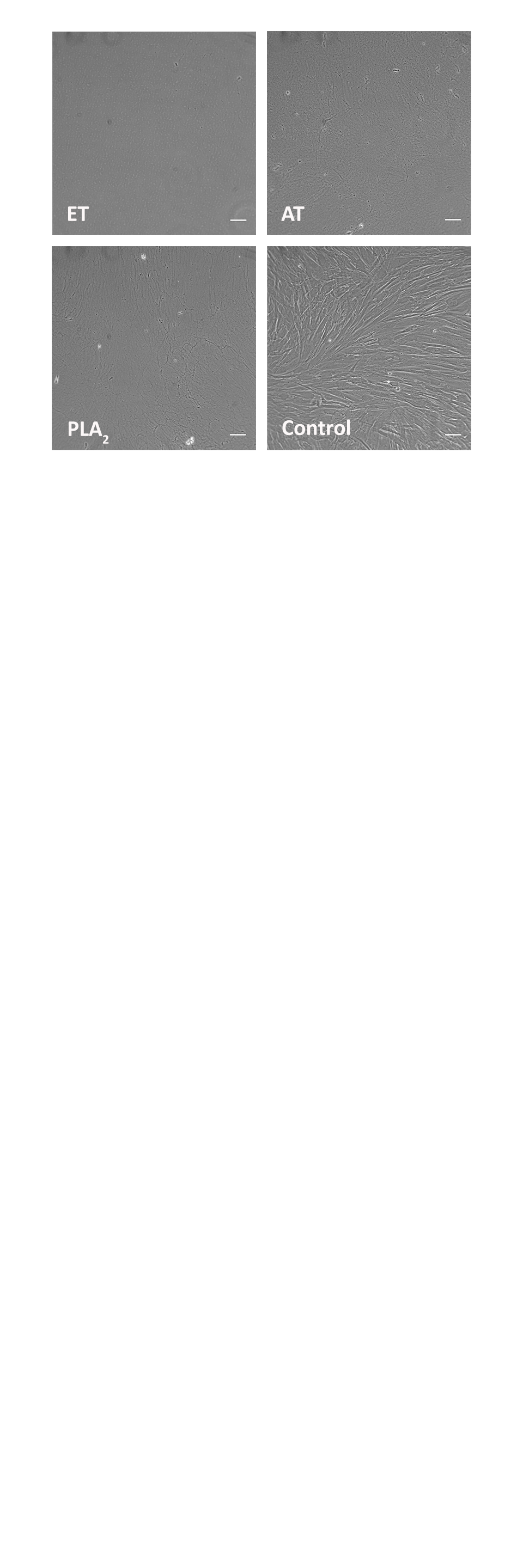
**

**Figure S4: Decellularised dermal fibroblast-derived ECM prepared using EDTA, ammonia hydroxide (AH) or phospholipase A_2_ (PLA_2_).** Images were obtained using phase contrast microscopy. The HDF were grown with MMC for seven days and decellularised as indicated. The control was HDF before decellularisation. Scale bars are 100 µm.

**
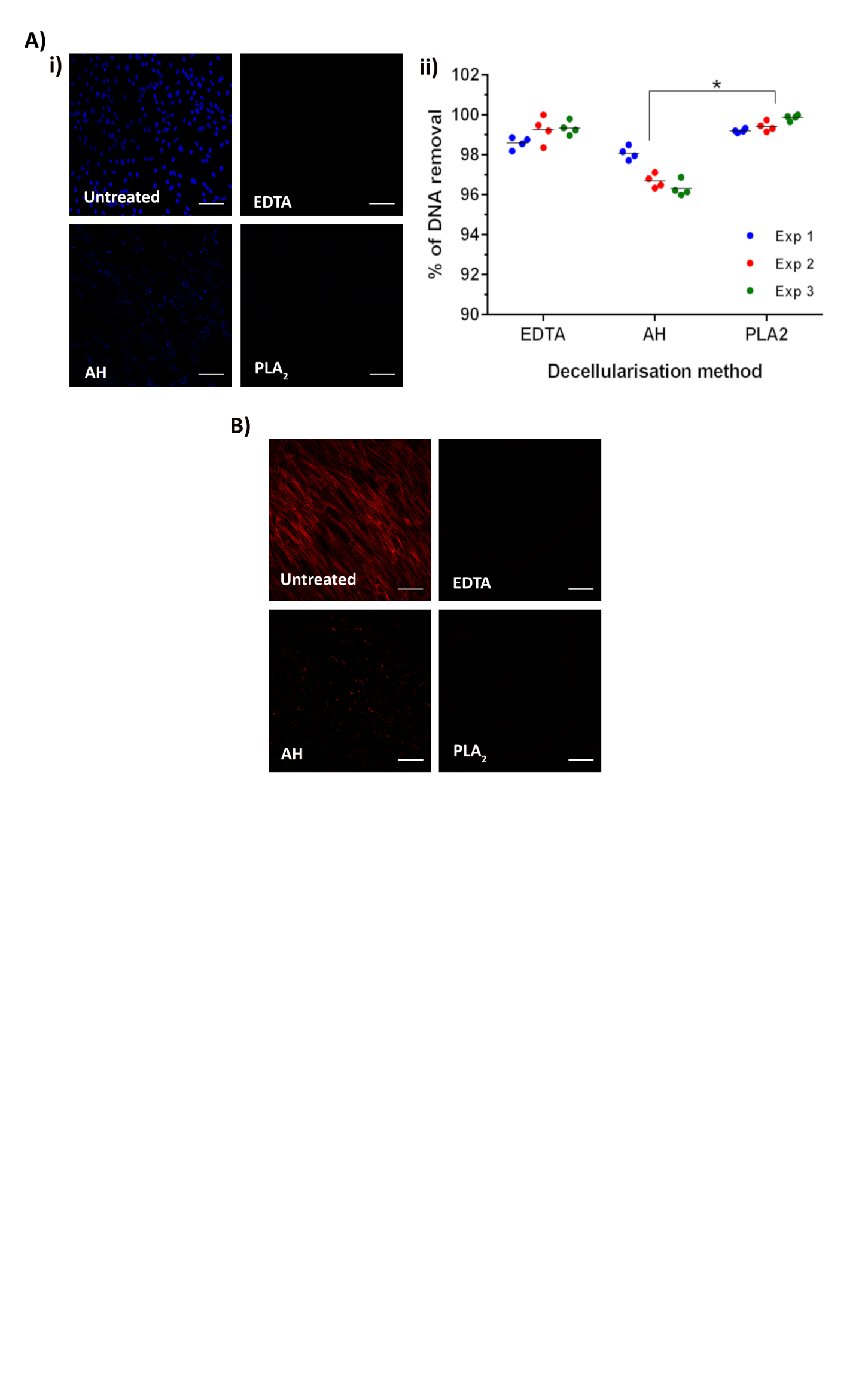
**

**Figure S5: Efficacy of methods for decellularisation of HDF matrices.**

**A)** Efficacy of decellularisation methods for removing nuclear components. i) DAPI staining of the variously decellularised ECM and untreated HDF control. HDF were grown with MMC for seven days then decellularised by the method indicated. Images were obtained using fluorescence microscopy. Scale bars are 100 µm. ii) Quantification of DNA removed after decellularisation. The CyQuant dye was used to measure the DNA present following decellularisation and these fluorescent intensity values were subtracted from the fluorescent intensity of the untreated control to allow calculation of percent of DNA removed. Data from three experiments are shown, and mean values are indicated by a line. ** = p<0.01.

**B)** Efficacy of different decellularisation treatments for removing cytoskeletal components. Phalloidin staining of the variously decellularised ECM and untreated HDF control cell layer for polymerised actin. Images were obtained using fluorescence microscopy. Scale bars are 100 µm.

**
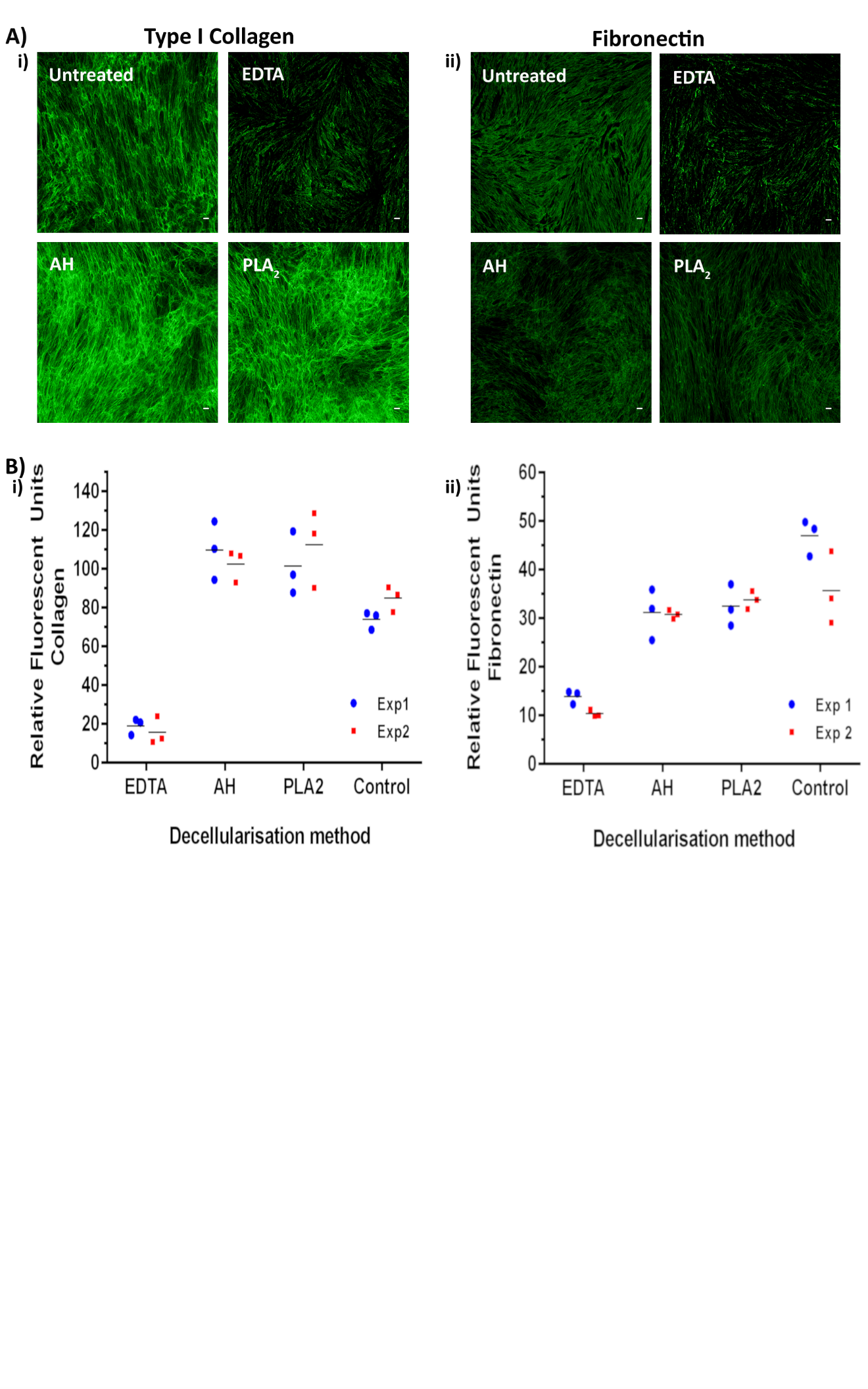
**

**Figure S6: Quantities of the deposited ECM after decellularisation.**

**A)** Representative images of ECM obtained using different decellularisation treatments: EDTA, ammonia hydroxide (AH) & phospholipase A_2_ (PLA_2_). The acellular ECM were immunostained using antibodies recognizing either type I collagen or fibronectin. Scale bars are 100 µm.

**B)** Quantification of fluorescence intensity of type I collagen (i) and fibronectin (ii) immunostaining after decellularisation. Data for two experiments are shown, and mean values are indicated by a line.

**
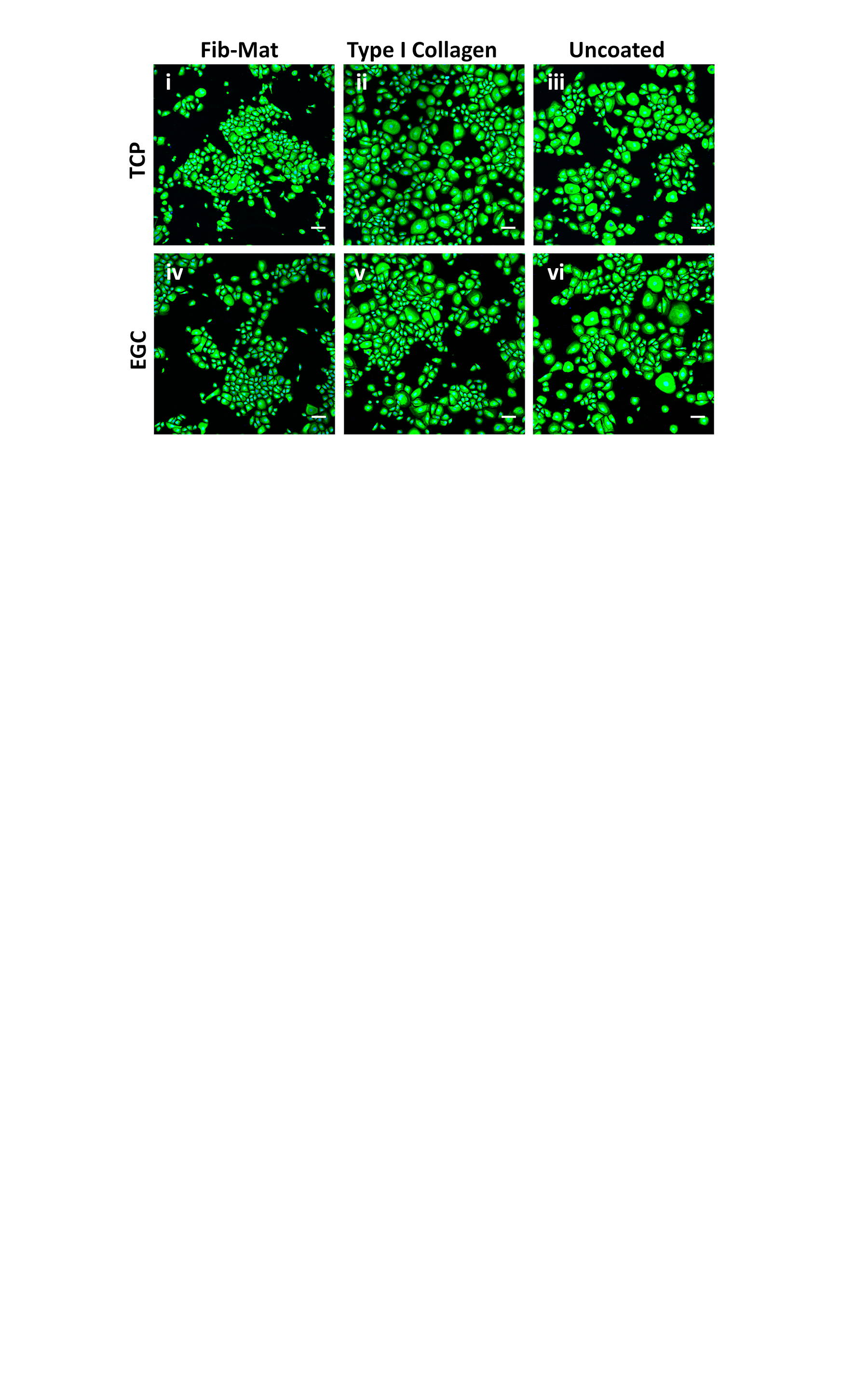
**

**Figure S7: Comparison of tissue culture plastic (TCP) and etched glass coverslips (EGC) as a platform for keratinocyte growth.**

Keratinocytes grown on either EGC or TCP coated with either Fib-Mat (i,iv), type I collagen (3ug/cm2: ii,iv) or were uncoated (plain: iii, vi). Keratinocytes were cultured for 3 days in DKSFM then fixed with acetone: methanol (1:1) and immunostained for K14. The secondary antibody was an anti-mouse IgG Alexa Fluor 488-conjugated antibody (Green). Nuclei were stained with DAPI (Blue). Scale bars are 100μm.
